# Neuronal excitability and parameter variability in the Hodgkin-Huxley model

**DOI:** 10.1371/journal.pcbi.1014458

**Published:** 2026-06-29

**Authors:** Alon Korngreen

**Affiliations:** 1 The Leslie and Susan Gonda Interdisciplinary Brain Research Center, Bar-Ilan University, Ramat Gan, Israel; 2 The Mina and Everard Goodman Faculty of Life Sciences, Bar-Ilan University, Ramat Gan, Israel; Ernst-Strungmann-Institut, GERMANY

## Abstract

Biophysically detailed neuron models are often built as a one-way pipeline in which voltage-clamp data are reduced to a single set of best-fit channel parameters, which are then combined into a deterministic spiking model. This practice discards experimentally observed scatter and fitting uncertainty, obscuring the mechanisms by which robustness and degeneracy arise in excitable systems. Here, I reintroduce fitted-parameter uncertainty into the Hodgkin-Huxley model and embed uncertainty and global sensitivity analysis into model construction. I digitized sodium and potassium rate-constant data from the original Hodgkin and Huxley figures and used bootstrap resampling to estimate uncertainty in the fitted voltage-dependent kinetic parameters. I then propagated these uncertainty estimates through a spatially extended squid axon cable model using large-scale Monte Carlo simulations, in which each sample defined a complete set of kinetic, conductance, passive, and structural parameters. At the channel level, first-order Sobol sensitivity indices revealed that all kinetic parameters contribute to output variance in a strongly time-dependent manner, with distinct parameters controlling transient and steady-state behavior for potassium and sodium conductances. At the level of neuronal excitability, the simulations produced a heterogeneous population of firing behaviors, including non-firing, phasic, regular, and spontaneous activity. Across stimulus amplitudes, the dominant firing mode was a single spike at stimulus onset, consistent with the physiological role of the squid giant axon in rapid escape behavior. The canonical 1952 Hodgkin-Huxley parameter set fell within the regularly firing minority subpopulation, rather than representing a unique or dominant solution. In the phasic subpopulation, action potential propagation and conduction velocity varied widely yet remained within experimental ranges. Finally, global sensitivity analysis during spiking showed uniformly small first-order Sobol indices but large total-order indices, indicating that excitability is governed primarily by strong interactions among all parameters rather than by any subset. Together, these results support reframing the Hodgkin-Huxley model as an experimentally constrained ensemble of behaviors rather than a single privileged parameter set, with physiologically relevant firing patterns emerging from structured regions of the parameter space.

## Author summary

Neurons are typically modeled by collapsing noisy ion-channel data into a single best-fit parameter set. I asked what happens to the classic Hodgkin-Huxley model of the squid giant axon when that uncertainty is preserved instead. Bootstrap resampling of the original 1952 rate-constant data, propagated through hundreds of thousands of cable simulations, produces a structured population of firing behaviors. Three results stand out. The dominant response is a single action potential rather than the textbook train of spikes, consistent with the axon’s role in escape behavior. The canonical 1952 parameter set represents a minority of regularly firing cases rather than the population’s typical behavior. And no single parameter controls spiking on its own; excitability emerges from interactions among many parameters. My results thus suggest that the Hodgkin-Huxley model is most informative not as a privileged parameter set but as an experimentally constrained ensemble of behaviors, and that biophysical modeling should estimate parameter uncertainty from data, build ensembles, and analyze them before drawing conclusions from any single model.

## Introduction

A primary approach to understanding the computational properties of neurons is the development of mathematical and computational models [1–7]. These models range from abstract representations that treat neurons as point-like integrators to biologically detailed simulations that incorporate neuronal morphology, membrane conductances, and intracellular biophysics [7–10], and they have become increasingly feasible through advances in experimental techniques, computational resources, and optimization methods [11–14]. Yet this progress has exposed a persistent methodological problem: as models become more detailed, parameters inherited from experiments are often treated as fixed, even though the measurements from which they are derived are subject to variability, uncertainty, and model-dependent assumptions [15].

The origins of biologically detailed conductance-based modeling trace back to the seminal work of Hodgkin and Huxley, whose formulation of action-potential generation in the squid giant axon laid the groundwork for the field [16]. Their framework established a systematic path from voltage-clamp recordings to rate constants, from rate constants to voltage-dependent equations, and from these equations to a spiking neuron model. Despite its enduring influence, this formalism functions as a reductionist one-way pipeline: each step reduces complexity by fitting variable data to a small set of parameters, while uncertainty in the underlying measurements is compressed into point estimates. The cost of this compression is particularly acute for channel kinetics. Once voltage-clamp data are fit to a small number of rate-constant parameters, those parameters are treated as fixed properties of the model, and uncertainty in the original measurements is not carried forward. The resulting model may reproduce a canonical firing pattern, but it cannot, by itself, reveal whether that pattern is robust, typical, or a member of a broader family of possible behaviors.

This issue is distinct from the well-established study of ion-channel conductance degeneracy. Multiple lines of work have shown that similar neuronal or network activity can arise from many distinct combinations of maximal conductances and other cellular parameters [17–27]. The database approach of Prinz, Marder, and colleagues was particularly influential in establishing that parameter degeneracy is a feature, not an artifact, of conductance-based models [12,17,18,20,26,28–30], with morphological variability and heterogeneous channel distributions further enlarging this equivalence class. More recently, simulation-based inference has provided a principled framework for moving beyond single best-fit parameter sets, using likelihood-free Bayesian inference and neural density estimation to recover posterior distributions over biophysical parameters [31,32]. These methods are flexible with respect to which parameters are treated as free. At the level of integrated Hodgkin-Huxley-type spiking models, however, channel kinetics are typically inherited as a prescribed model structure [33], while inference is performed over conductances and a smaller set of additional membrane or adaptation parameters [31,32]. Recent differentiable simulators have begun to extend related inference approaches to spatially extended neurons [14].

Variability in kinetic parameters, therefore, remains underexploited at two distinct stages of the modeling pipeline. At the construction stage, channel kinetics are often fit once, frozen, and used as a fixed scaffold on which conductance densities and other parameters are tuned, whether by hand, by stochastic optimization algorithms [7,22,23,34–43], or by inference methods conditioned on selected features. At the analysis stage, variability and sensitivity analyses are applied after a model has already been constructed, rather than treated as part of model construction itself. The canonical Hodgkin-Huxley model is a natural place to address this gap. Although the original voltage-clamp traces are unavailable, so that full biological variability in the squid giant axon cannot be recovered, the published Hodgkin-Huxley figures and tables do provide experimentally grounded information about the uncertainty in the fitted rate equations. This uncertainty reflects a mixture of experimental scatter, digitization error, fitting uncertainty, and assumptions imposed by the chosen functional forms, and should not be interpreted as biological variability alone. It can, however, be used to ask a concrete question: how does uncertainty that is already visible in the published rate-constant data propagate through the canonical model of excitability?

I therefore ask a narrower question than whether neuronal excitability is generally a population-level property, a point that previous work has already established convincingly [17,19–21,27–29,44,45]. Given experimentally grounded uncertainty in the voltage-dependent kinetic parameters of the canonical Hodgkin-Huxley model, what is the structure of the resulting population of behaviors, and which features of the canonical model survive that uncertainty? Does the firing pattern produced by the canonical 1952 parameter set [16] represent a typical outcome of the Hodgkin-Huxley framework, or a special case within a broader landscape? And to what extent is excitability in this population governed by individual parameters rather than by interactions among them?

To address these questions, I explicitly reintroduced parameter uncertainty into the Hodgkin-Huxley modeling pipeline and embedded uncertainty and sensitivity analysis into model construction. I first estimated uncertainty in the sodium and potassium channel kinetic parameters from the original Hodgkin-Huxley figures [16] using bootstrap resampling, yielding empirical distributions for each fitted parameter. I then propagated this uncertainty through a spatially extended Hodgkin-Huxley cable model of the squid giant axon using large-scale Monte Carlo simulations, in which each sample defined a complete model with kinetic parameters drawn from the bootstrap-derived distributions and conductance, passive, and structural parameters varied over ranges derived from the Hodgkin-Huxley framework. Finally, I applied a global variance-based sensitivity analysis using Sobol decomposition to both voltage-clamp simulations of isolated conductances and action-potential generation in the cable model, making it possible to ask how individual parameters and their interactions contribute to channel-level behavior and to neuronal excitability.

This combined analysis yields three main results. First, the inferred parameter uncertainties are substantial and parameter-specific, reflecting both scatter in the original published data and redundancies in the fitted rate-constant equations. Second, propagating these uncertainties through the cable model produces a heterogeneous population in which the dominant firing mode is a single action potential at stimulus onset, consistent with the physiological role of the squid giant axon in rapid escape behavior; the canonical 1952 parameter set falls within the regularly firing minority and closely matches the mean firing-rate behavior of that subpopulation, rather than representing the dominant solution. Third, sensitivity analysis shows that individual kinetic parameters exert pronounced first-order effects at the channel level, but during spiking, first-order Sobol indices are small while total-order indices are large. Excitability is therefore governed primarily by interactions among parameters rather than by independent effects of any single one. Kinetic parameters that are often frozen during conductance fitting remain consequential in the integrated system, supporting a reframing of the Hodgkin-Huxley model as an experimentally constrained ensemble of behaviors rather than a single privileged parameter set.

## Methods

### Ethics statement

No human or animal subjects were used in this study.

### Biophysical model of an excitable cable

Simulations of neuronal excitability, unless stated otherwise, used a spatially extended Hodgkin-Huxley-type cable model. The axon was represented as a one‑dimensional cylindrical cable of length 10 cm and radius 0.025 cm, electrically discretized into 80 isopotential compartments. Each compartment contained voltage‑gated sodium and potassium conductances as well as a passive leak conductance. The membrane potential *V(x,t)* evolved according to the cable equation with active ionic currents. The membrane dynamics were governed by:


$$\begin{array}{c} C_{m}\frac{\partial V}{\partial t}=\frac{\pi a^{\text{2}}}{R_{a}}\frac{\partial^{\text{2}}V}{{\partial x}^{\text{2}}}-I_{ion}(V,m,h,n)+I_{ext}(x,t) \end{array}$$

Here, *C*_*m*_ is the membrane capacitance per unit area (ranges defined in Table 1), *a* is the cable radius (0.025 cm in all simulations), *R*_*a*_ is the axial resistivity (35.4 Ω·cm in all simulations), *I*_*ion*_ is the sum of ionic currents, and *I*_*ext*_ represents externally injected current. The total ionic current density was the sum of fast sodium, delayed‑rectifier potassium, and leak currents:

**Table 1:** Parameters of the Hodgkin-Huxley model and the ranges used in simulations. The original value from the Hodgkin-Huxley manuscript is provided for comparison. The kinetic parameters obtained from the bootstrap analysis are reported with the standard deviation from that analysis (and also in Eqs 1–6). Maximal conductance, reversal potentials, and membrane capacitance were extracted from the Hodgkin-Huxley manuscript [16].

Name	Units	Original Value	Fitted Value	Standard Deviation
${\overset{―}{g}}_{K}$	mS/cm^2^	36	–	6
$E_{K}$	mV	-77	–	2
$A_{\alpha ,n}$	1/(mV•ms)	0.01	0.0092	0.0004
${V\text{1}/\text{2}}_{\alpha ,n}$	mV	55	59.0	2.8
$z_{\alpha ,n}$	mV	10	6.9	1.8
$A_{\beta ,n}$	1/ms	0.055	0.066	0.003
$z_{\beta ,n}$	mV	80	100.0	13.5
${\overset{―}{g}}_{Na}$	mS/cm^2^	120	–	50
$E_{Na}$	mV	45	–	6
$A_{\alpha ,m}$	1/(mV•ms)	0.1	0.103	0.008
${V\text{1}/\text{2}}_{\alpha ,m}$	mV	40	39.1	4.1
$z_{\alpha ,m}$	mV	10	6	2
$A_{\beta ,m}$	1/ms	0.11	0.12	0.08
$z_{\beta ,m}$	mV	18	15.9	4.2
$A_{\alpha ,h}$	1/ms	0.0027	0.0044	0.0014
$z_{\alpha ,h}$	mV	20	22.1	2.1
$A_{\beta ,h}$	1/ms	1	0.96	0.10
${V\text{1}/\text{2}}_{\beta ,h}$	mV	35	34.8	5.7
$z_{\beta ,h}$	mV	10	11.8	4.7
$C_{m}$	µF/cm^2^	0.91	–	0.2
${\overset{―}{g}}_{L}$	mS/cm^2^	0.3	–	0.1
$E_{L}$	mV	-53	–	4


$$\begin{array}{c} I_{ion}={\overset{―}{g}}_{Na}m^{\text{3}}h\left(V-E_{Na}\right)+{\overset{―}{g}}_{K}n^{\text{4}}\left(V-E_{K}\right)+{\overset{―}{g}}_{L}\left(V-E_{L}\right) \end{array}$$

Where ${\overset{―}{g}}_{Na}$, ${\overset{―}{g}}_{K}$, and ${\overset{―}{g}}_{L}$ are maximal conductances and $E_{Na}$, $E_{K}$, and $E_{L}$ are reversal potentials. Gating variables *m, h*, and *n* followed first‑order voltage‑dependent kinetics:


$$\begin{array}{c} \frac{dm}{dt}=\alpha_{m}(\text{1}-m)-\beta_{m}m \end{array}$$

$$\begin{array}{c} \frac{dh}{dt}=\alpha_{h}(\text{1}-h)-\beta_{h}h \end{array}$$

$$\begin{array}{c} \frac{dn}{dt}=\alpha_{n}(\text{1}-n)-\beta_{n}n \end{array}$$

The transition rates were defined using standard Hodgkin-Huxley functional forms, with parameters controlling amplitudes, slopes, and voltage offsets (see Results below). All rate expressions were evaluated with numerical safeguards to avoid singularities, and gating variables were constrained to the interval [0,1].

### Spatial discretization and boundary conditions

The cable was discretized using a second‑order finite‑difference approximation of the axial second derivative. Sealed‑end boundary conditions were imposed at both ends of the cable, enforcing zero axial current flow. This resulted in a system of 320 coupled ordinary differential equations representing membrane voltage and gating dynamics across space.

An external current was injected into a single compartment located 0.4 cm from the cable’s sealed end. The injected current was specified as a total current and converted into a current density by dividing by the membrane surface area of the stimulated segment. The stimulus consisted of a square pulse with linear onset and offset ramps, beginning at 10 ms and ending at 80 ms. Voltage responses were recorded both at the injection site and at a distal location 8 cm away.

For each parameter set, the initial condition was chosen as the equilibrium (resting) state of the single–compartment Hodgkin-Huxley model corresponding to those parameters. The resting potential V_rest_ was defined as the voltage at which the total ionic current vanished when all gating variables were at steady state. In practice, I numerically found the solution to the algebraic function:


$$\begin{array}{c} F(V)\equiv I_{Na}\left(V,m_{\infty },h_{\infty }\right)+I_{k}\left(V,n_{\infty }\right)+I_{L} \end{array}$$

The steady-state gating variables were defined as: x_∞_(V)=α_x_(V)/(α_x_(V)+β_x_(V)) for x ∈ {m, h, n}. The equilibrium voltage was obtained using a damped Newton iteration with a finite-difference derivative approximation. This approach modified the standard method in three key ways. Step Damping: Instead of taking the full Newton step, only 30% of the step was applied. This smaller, more conservative update prevents the solver from jumping too far in any single iteration. Step Limiting: The maximum change in voltage per iteration was capped at 5 mV. This prevents large, unstable jumps when the local derivative is very small. Boundary Constraints: The voltage was strictly bounded within a physiological range (-100 mV to +50 mV) at each step. These modifications ensure that the algorithm remains stable and reliably converges to the correct physiological resting potential, even when starting from a broad range of initial guesses or exploring a wide parameter space in Monte Carlo simulations. Once V_rest_ was found, all compartments were initialized to this voltage and the corresponding steady-state gating values, yielding a spatially uniform resting state.

### Numerical integration and implementation

All simulations were implemented in Python (3.12.4) using NumPy (2.2.1) and JAX (0.8.1 [46]) for numerical computation, and the Diffrax library (0.7.0 [47]) for ordinary differential equation integration. The coupled system was integrated using an adaptive fifth‑order Runge–Kutta method with relative and absolute tolerances of 10 ⁻ ⁵ and 10 ⁻ ⁶, respectively.

Simulations were run for 100 ms of biological time, and membrane potentials were recorded at fixed intervals of 0.1 ms. To enable large‑scale parameter exploration, simulations were vectorized and parallelized using JAX’s just‑in‑time compilation, automatic vectorization, and parallel mapping across CPU cores.

**Fig 1:**
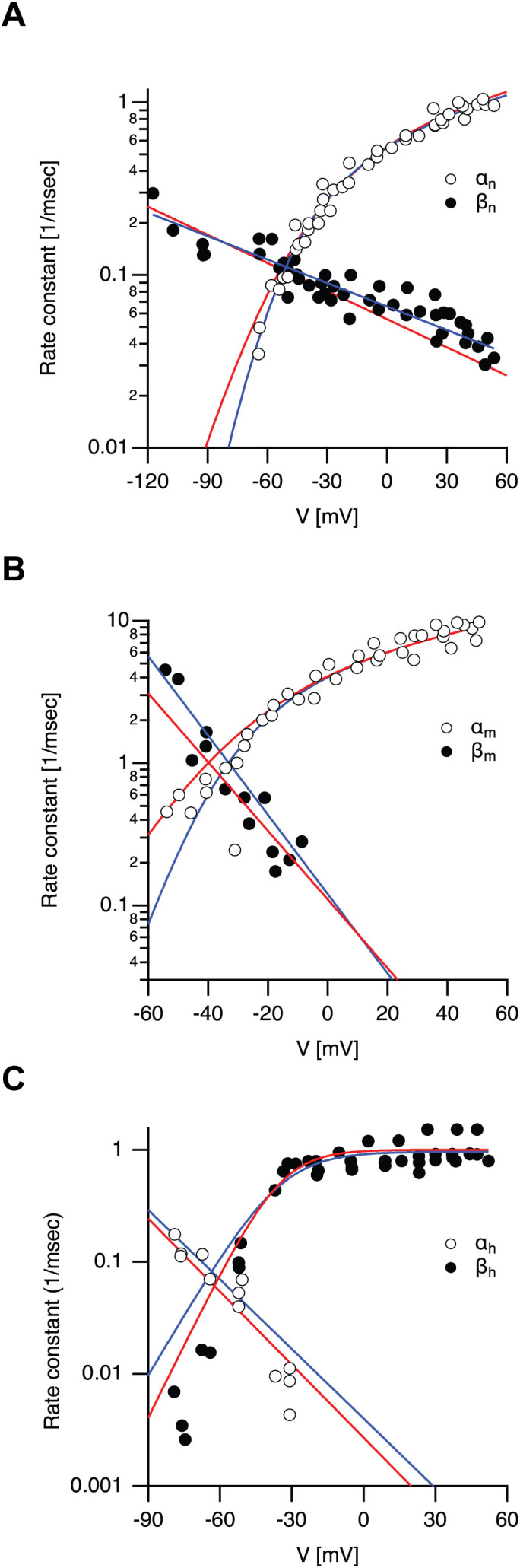
Refitting the rate constants of the Hodgkin-Huxley model. A, the forward (white circles) and backward (black circles) rate constants for the potassium conductance, extracted from the Hodgkin-Huxley paper. The original Hodgkin-Huxley-calculated rate constants are plotted in red, and the new fit from the bootstrap analysis is plotted in blue. B, the same for the rate constants defining the activation of the sodium conductance. C, the same for the rate constants defining the inactivation of the sodium conductance.

Model parameters were sampled using simple Monte Carlo sampling over the ranges obtained in Fig 1 and presented in Table 1. The sampled parameters included maximal conductances, reversal potentials, membrane capacitance, and all kinetic parameters governing sodium and potassium channel gating. The number of parameter sets sampled is indicated in the results and figure legends. Several safeguards were implemented to ensure numerical stability and to prevent unphysical states during large-scale Monte Carlo simulations. Rate expressions that contain potentially ill-conditioned terms of the form in the denominator were evaluated using limiting expressions when the denominator approached zero. All gating variables were constrained by clipping to the interval [0,1] at every solver evaluation.

As detailed above, the resting potential was computed using a damped Newton iteration to solve the steady-state current-balance equation. Newton updates were bounded in magnitude to avoid instability in poorly conditioned parameter regimes, and the finite-difference estimate of the derivative was regularized by enforcing a small minimum magnitude. Although the production simulations used an adaptive solver, this bound provided a robust initial step size for the stiffest parameter combinations encountered in the Monte Carlo ensemble.

The overall simulation workflow was as follows. For each Monte Carlo draw, a complete parameter set was generated by sampling the kinetic, conductance, reversal-potential, leak, and capacitance parameters from the ranges listed in Table 1. That same parameter set was applied uniformly to all compartments of the cable. The resting potential and steady-state gating variables corresponding to that parameter set were then computed, the cable was initialized to this spatially uniform equilibrium state, and the response to current injection was simulated. The resulting voltage trace at the injection site was then classified as non-firing, phasic, regular, or spontaneous according to the spike-detection criteria described below.

### Bootstrap parameter estimation

Parameter uncertainties for each voltage-dependent rate constant of the Hodgkin-Huxley model were estimated by bootstrap resampling. The procedure was performed independently for each of the six rate constants ($\alpha_{m}$, $\beta_{m}$, $\alpha_{h}$, $\beta_{h}$, $\alpha_{n}$, $\beta_{n}$). For each rate constant, the digitized data points from the original paper [16] were resampled with replacement 4,000 times. Each bootstrap dataset was refitted using the same nonlinear least-squares procedure (Python, scipy.optimize.curve_fit) with the same model and initial parameter guesses as the original fit, yielding an empirical distribution of 4,000 parameter estimates per rate constant. The mean and standard deviation of each distribution were taken as the parameter estimate and its uncertainty, respectively. A fixed random seed was used for reproducibility.

### Sensitivity analysis

Sensitivity analysis evaluates how uncertain parameters influence the variability of model outputs. Numerous sensitivity measures are available [48–53]. In this study, I employed a global variance-based sensitivity analysis that calculates Sobol sensitivity indices, a well-established approach [54]. This global and non-intrusive method enables investigating interactions among model parameters [50]. To quantify how variability in Hodgkin-Huxley model parameters influences neuronal excitability, I performed a global variance-based sensitivity analysis. This approach explicitly treats model parameters as random variables and evaluates how their uncertainty propagates to variability in model outputs. Sensitivity analysis was embedded directly into the simulation pipeline rather than applied post hoc to a single optimized parameter set.

Each sampled parameter set was used to simulate the neuronal response to a current injection. Formally, a model output $Y\;=\;f(Q_{\text{1}},\;Q_{\text{2}},\;\ldots ,\;Q_{p})$ was defined as a function of *p* uncertain parameters *Q*_*i*_, including maximal conductances, reversal potentials, membrane capacitance, and kinetic parameters governing sodium and potassium channel gating. Sensitivity was quantified using Sobol variance decomposition.

The first-order Sobol sensitivity index for parameter *Q*_*i*_ was defined as:


$$\begin{array}{c} S_{i}=\frac{V[E\left[Y|Q_{i}\right]]}{V[Y]} \end{array}$$

$E\left[Y|Q_{i}\right]\;$represents the expected value of the output Y when the parameter Q_i_ is held constant. $V\left[E\left[Y|Q_{i}\right]\right]$ quantifies the variance explained by Q_i_ alone, and V[Y] is the variance of the output. The first-order Sobol sensitivity index indicates how much the model’s variance is expected to decrease when parameter Q_i_ is held constant. For example, if S_i_ = 0.3, then 30% of the output variance is explained by Q_i_ alone. The sum of all first-order Sobol sensitivity indices cannot exceed one and equals one only if there are no interactions between parameters [55].

To capture interaction effects between parameters, total-order sensitivity indices were also computed. The total sensitivity index for parameter *Q*_*i*_ was defined as:


$$\begin{array}{c} S_{T}=\frac{V[E\left[Y|Q_{-i}\right]]}{V[Y]} \end{array}$$

$E\left[Y|Q_{-i}\right]\;$represents the expected value of the output Y when all parameters, except Q_i,_ are held constant. $V\left[E\left[Y|Q_{-i}\right]\right]$ quantifies the variance explained by all variables except Q_i_. For example, if S_Ti_ = 0.5, then *Q*_*i*_ (including its interactions with other variables) explains 50% of the output variance.

Sobol indices were estimated using Monte Carlo sampling based on Saltelli’s extension of the Sobol method [49]. Unless stated otherwise, kinetic parameters were sampled independently from Gaussian distributions centered on the bootstrap mean and truncated at mean ± 2 SD. For parameters whose lower bounds would become nonphysical, the lower bounds were adjusted as described below. All simulations used identical solver tolerances, temporal discretization, and spike-detection criteria to ensure numerical consistency across the ensemble. Sensitivity indices were computed across the membrane potential response, allowing comparison of parameter influence during action-potential generation and during the sustained post-spike/depolarized state. All simulations were carried out using custom Python code and the SALib sensitivity analysis library (1.5.1 [56]). The code is available at https://github.com/alon67/HodgkinHuxleyVariability.

## Results

I first reconstructed uncertainty in the voltage-dependent rate equations that define the Hodgkin-Huxley sodium and potassium conductances. In the original Hodgkin-Huxley workflow, voltage-clamp currents were reduced to rate constants, and those rate constants were then fit as functions of voltage. Because the original voltage-clamp traces are unavailable, I extracted the published rate-constant data from the Hodgkin-Huxley figures and refit the voltage-dependent equations.

I extracted the figures from the PDF of the Hodgkin-Huxley manuscript [16], enlarged them on a computer monitor, calibrated the figure axes to the screen coordinates in Adobe Photoshop, and then measured each data point by hand. To verify this extraction method, I compared the obtained values to the sample data provided in the Hodgkin-Huxley paper [16]. This was done for the six rate constants defining the Hodgkin-Huxley model. The voltage axis in the original Hodgkin-Huxley paper was relative to the resting membrane potential. I converted this to absolute membrane potential by adding a resting potential of -65 mV to the data and to the Hodgkin-Huxley equations. The original Hodgkin-Huxley equations describing the voltage dependence of the rate constants for potassium conductance are therefore:


$$\begin{array}{c} \alpha_{n}=\frac{\text{0}.\text{01}(V+\text{55})}{\left(\text{1}-exp\left(-\frac{\left(V+\text{55}\right)}{\text{10}}\right)\right)} \end{array}$$

$$\begin{array}{c} \beta_{n}=\text{0}.\text{125}\text{exp}\left(-\frac{\left(V+\text{65}\right)}{\text{80}}\right) \end{array}$$

It is important to note that the equation describing *β*_*n*_ contains a fitting redundancy, which can lead to multiple equivalent parameterizations during curve fitting.

Thus, it is better to simplify this rate constant to:


$$\begin{array}{c} \beta_{n}=\text{0}.\text{055 }\text{exp}\left(-\frac{V}{\text{80}}\right) \end{array}$$

**Fig 2:**
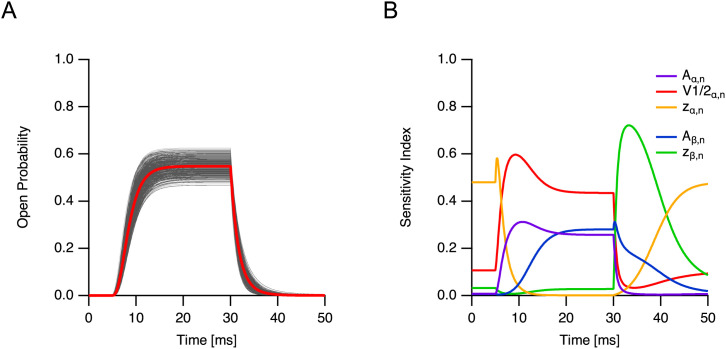
Sensitivity analysis of the potassium conductance. A, Simulations using different parameter sets of a voltage-clamp experiment in which the membrane potential was stepped from -80 to -10 mV. 500 simulations of the conductance open probability are shown in gray as a function of time, with the population average in red. B, First-order sensitivity indices, calculated from 98,304 Monte Carlo simulations (N = 8192 with Saltelli sampling), for the five kinetic parameters defining the voltage dependence of the potassium conductance are plotted as a function of time.

**Fig 3:**
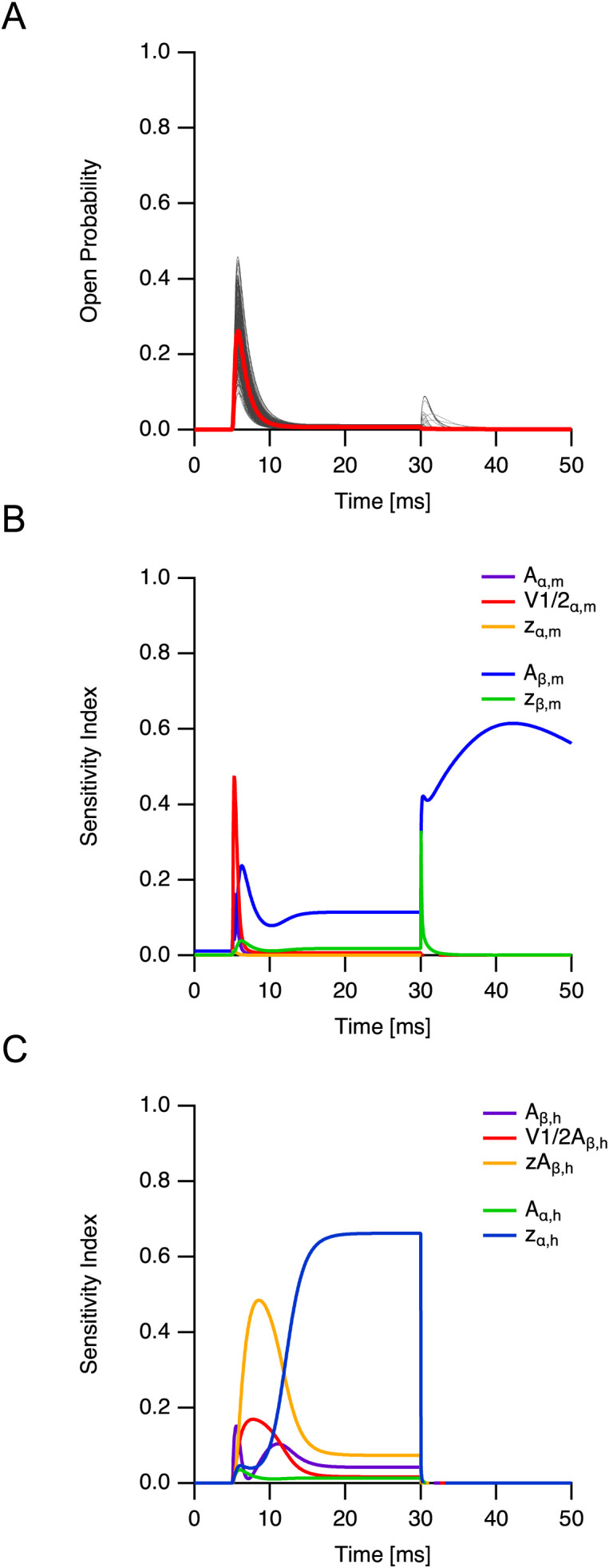
Sensitivity analysis of the sodium conductance. A, Simulations using different parameter sets of a voltage-clamp experiment in which the membrane potential was stepped from -80 to -10 mV. 500 simulations of the conductance open probability are shown in gray as a function of time, with the population average in red. B, First-order sensitivity indices, calculated from 90,112 Monte Carlo simulations (N = 4096 with Saltelli sampling), for the five kinetic parameters defining the voltage dependence of the sodium conductance activation process are plotted as a function of time. C, First-order sensitivity indices for the five kinetic parameters defining the voltage dependence of the sodium conductance inactivation process are plotted as a function of time.

In what follows, I retain the original Hodgkin-Huxley functional forms but treat each numerical constant as a fitted parameter with a systematic name (Table 1). In α_*n*_, the amplitude prefactor becomes *A*_*α,n*_, the voltage offset becomes *V1/2*_*α,n*_, and the slope factor in the denominator becomes *z*_*α,n*_; in β_*n*_, the amplitude becomes *A*_β*,n*_ and the slope factor in the exponent becomes *z*_β*,n*_. The same convention applies to the sodium activation rate constants (m-gate: *A*_*α,m*_, *V1/2*_*α,m*_, *z*_*α,m*_, *A*_β*,m*_, *z*_β*,m*_) and to the sodium inactivation rate constants (h-gate: *A*_*α,h*_, *z*_*α,h*_, *A*_β*,h*_, *V1/2*_β*,h*_, *z*_β*,h*_). These fifteen parameters are the quantities estimated by bootstrap below; they are listed with their original Hodgkin-Huxley values and refit means in Table 1, and they are the axes of the sensitivity decompositions reported in Figs 2 and 3.

**Fig 4:**
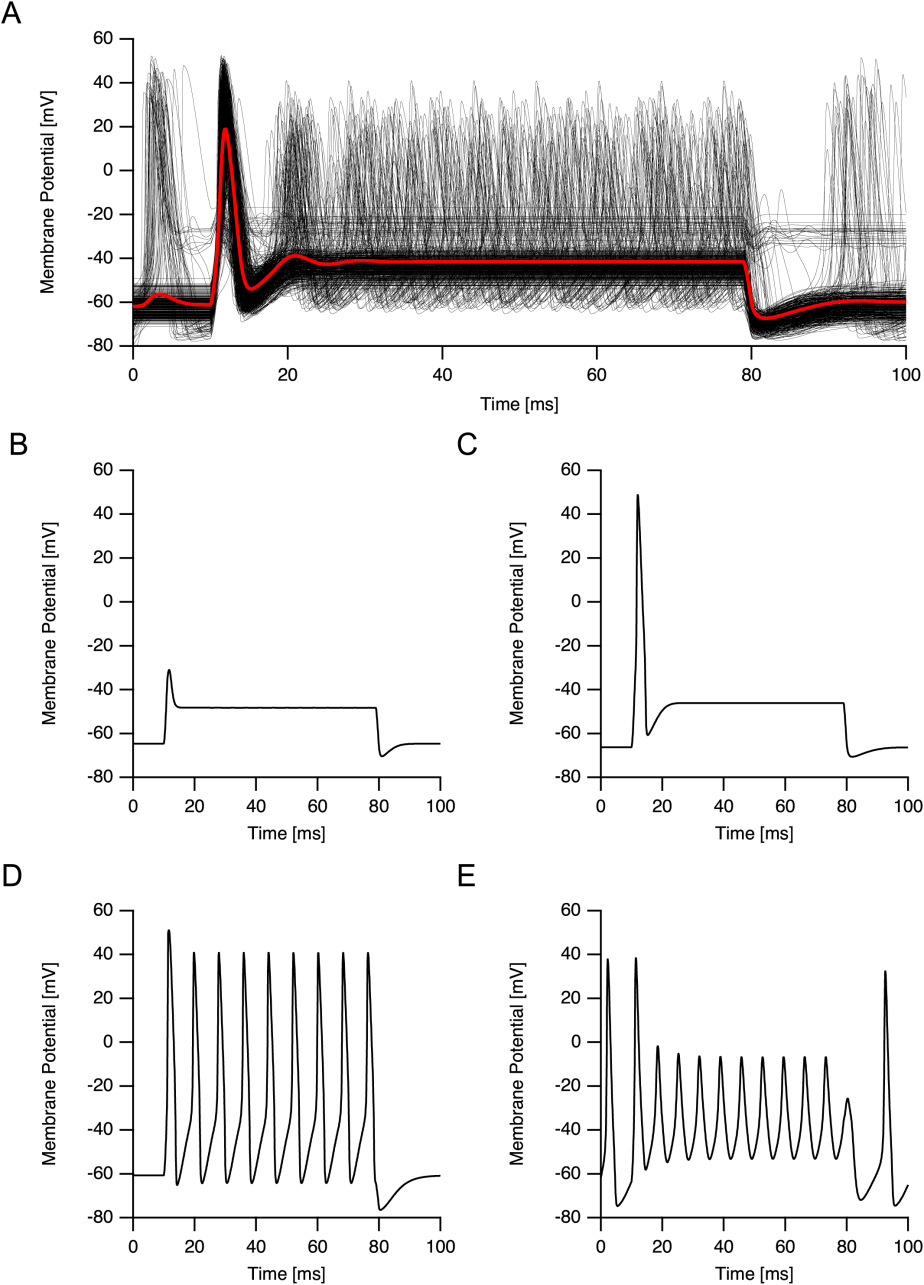
Population structure of firing behaviors in the Hodgkin-Huxley axon model. A, Five hundred representative membrane potential traces recorded at the site of current injection during a 70-ms square current pulse, randomly selected from the entire Monte Carlo ensemble of 300,000 parameter sets. Individual simulations are shown in gray, and the population-average membrane potential is overlaid in red. B–E, Based on their voltage responses, simulations were classified into four distinct firing phenotypes: B, nonfiring responses showing only subthreshold depolarization, C, phasic responses generating a single action potential at stimulus onset, D, regular firing responses producing multiple action potentials during the stimulus, and E, spontaneous firing responses in which action potentials occurred in the absence of injected current.


To estimate the parameter uncertainties in Eqs. 1 and 2, I digitized the potassium conductance rate-constant data from Fig 4 of the original Hodgkin-Huxley paper. Parameter uncertainty was quantified using the bootstrap procedure described in the Methods. Specifically, the extracted data points were resampled with replacement to generate bootstrap datasets, which were refitted using the same nonlinear least-squares procedure as in the original fit. This yielded an empirical distribution for each fitted parameter, from which the mean value and standard deviation were computed and used as estimates of the parameter value and its variance. Because each rate constant was fitted separately, any correlations among parameters reflected the local geometry of each rate equation, and parameters from different rate constants were largely uncorrelated. Parameter distributions and pairwise correlations for all rate constants defining the potassium conductance are shown in S1 Fig. The new rate constants, with the mean and standard deviation, are given in Eqs. 1 and 2, and the fit is shown in Fig 1A.

$$\begin{array}{c} \alpha_{n}=\frac{(\text{0}.\text{0092}\pm \text{0}.\text{0004})\left(V+(\text{59}.\text{0}\pm \text{2}.\text{8})\right)}{\left(\text{1}-exp\left(-\frac{\left(V+(\text{59}.\text{0}\pm \text{2}.\text{8})\right)}{\left(\text{6}.\text{9}\pm \text{1}.\text{8}\right)}\right)\right)} \end{array}$$ (1)

$$\begin{array}{c} \;\beta_{n}=(\text{0}.\text{066}\pm \text{0}.\text{003})\text{exp}\left(-\frac{V}{\left(\text{100}\pm \text{13}.\text{5}\right)}\right) \end{array}$$ (2)

The sodium conductance has four rate constants, two for activation and two for inactivation. For the activation process, the rate constants from the Hodgkin-Huxley model, corrected for a resting membrane potential of -65 mV are:


$$\begin{array}{c} \alpha_{m}=\frac{\text{0}.\text{1}(V+\text{40})}{\left(\text{1}-exp\left(-\frac{\left(V+\text{40}\right)}{\text{10}}\right)\right)} \end{array}$$

$$\begin{array}{c} \beta_{m}=\text{0}.\text{11 }\text{exp}\left(-\frac{V}{\text{18}}\right) \end{array}$$

Extracting the activation rate constants from Fig 7 in the Hodgkin-Huxley paper and refitting them using bootstrap to estimate parameter variation provided:


$$\begin{array}{c} \alpha_{m}=\frac{(\text{0}.\text{103}\pm \text{0}.\text{008})\left(V+(\text{39}.\text{1}\pm \text{4}.\text{1})\right)}{\left(\text{1}-exp\left(-\frac{\left(V+(\text{39}.\text{1}\pm \text{4}.\text{1})\right)}{\left(\text{6}\pm \text{2}\right)}\right)\right)} \end{array}$$ (3)

$$\begin{array}{c} \;\beta_{m}=(\text{0}.\text{12}\pm \text{0}.\text{08})\;\text{exp}\left(-\frac{V}{\left(\text{15}.\text{9}\pm \text{4}.\text{2}\right)}\right) \end{array}$$ (4)

The new rate constants with the mean fit and standard deviation are presented in Eqs. 3 and 4, and the fit is shown in Fig 1B. For inactivation, the rate constants from the Hodgkin-Huxley paper are:


$$\begin{array}{c} \alpha_{h}=\text{0}.\text{0027 }\text{exp}\left(-\frac{V}{\text{20}}\right) \end{array}$$

$$\begin{array}{c} \beta_{h}=\frac{\text{1}}{\left(\text{1}+exp\left(-\frac{\left(V+\text{35}\right)}{\text{10}}\right)\right)} \end{array}$$

As with the previous rate constants, the data points were extracted from the original Hodgkin-Huxley paper. Bootstrap was used to estimate the standard deviation of the curve fit of these data to Eqs. 5 and 6, as shown in Fig 1C. All the parameters appearing in Eqs. 1–6 are summarized in Table 1, which also contains systematic naming of all the parameters. Parameter distributions and pairwise correlations for all rate constants defining the sodium conductance are shown in S2 Fig.


$$\begin{array}{c} \;\alpha_{h}=(\text{0}.\text{0044}\pm \text{0}.\text{0014})\;\text{exp}\left(-\frac{V}{\left(\text{22}.\text{1}\pm \text{2}.\text{1}\right)}\right) \end{array}$$ (5)

$$\begin{array}{c} \;\beta_{h}=\frac{\left(\text{0}.\text{96}\pm \text{0}.\text{10}\right)}{\left(\text{1}+exp\left(-\frac{\left(V+(\text{34}.\text{8}\pm \text{5}.\text{7})\right)}{\left(\text{11}.\text{8}\pm \text{4}.\text{7}\right)}\right)\right)} \end{array}$$ (6)

Almost all 15 parameters in this bootstrap analysis followed a bell-shaped distribution (S1 and S2 Figs. along the diagonal). The two parameters controlling the backward rate of activation for the sodium conductance ($\beta_{m}$) were an exception, likely due to the small number of data points and large scatter in the original measurements (S2 Fig). Given this pattern, I decided to draw parameter values from Gaussian distributions for all Monte Carlo simulations. The upper and lower bounds of parameter sampling were set to the mean ± 2 SD for each parameter, according to Eqs. 1–6 and Table 1. This rule generated negative values of $A_{\beta ,m}$ due to the asymmetric shape of its distribution (S2 Fig); therefore, the lower bound for this parameter was set to10^-10^. The mean and standard deviation of the maximal conductances, reversal potentials, and membrane capacitance were obtained from the Hodgkin and Huxley paper [16]. Monte Carlo simulations in which parameter values were drawn from a uniform distribution yielded results similar to those obtained with parameter values drawn from a Gaussian distribution.

The pairwise scatter plots in S1 and S2 Figs reveal that parameter correlations are concentrated within individual rate constants (colored panels), while parameters from different rate constants are largely uncorrelated (black panels). This pattern is expected because each rate constant was bootstrapped independently: within a rate equation, parameters such as the amplitude and slope factor trade off against one another to reproduce the same voltage dependence, giving rise to structured joint distributions.

I used the results of this uncertainty analysis to assess parameter sensitivity in models of potassium and sodium conductances. I simulated each conductance under voltage-clamp conditions in a single compartment without spatial dimensions, and I computed the first-order Sobol’ sensitivity indices (Si) over time using Monte Carlo simulations. Fig 2A illustrates a voltage-clamp experiment where the membrane potential was stepped from -80 mV to -10 mV. The kinetic parameters defining Eqs. 1 and 2 were randomly sampled as defined above. To focus solely on kinetic sensitivity, I simulated changes in the channel’s open probability without altering its maximal conductance. The potassium conductance, defined by five kinetic parameters in the Hodgkin-Huxley model, each influenced the output variance uniquely over time (Fig 2B).

Similarly, I performed Monte Carlo simulations to assess how the parameters defining the sodium conductance, as described by Eqs. 3–6 and Table 1, affect its opening probability (Fig 3). Fig 3A shows a simulated voltage-clamp experiment in which the membrane potential was stepped from -80 mV to -10 mV. Randomly simulated traces are plotted, and the population average is overlaid in red. Ten kinetic variables define the sodium conductance in the Hodgkin-Huxley model. All of them displayed time-dependent sensitivity indices (Fig 3B and 3C). Most parameters related to conductance activation showed transient changes when the voltage was stepped from -80 to -10 mV (Fig 3B). As expected from the tenfold-slower inactivation process, the parameters controlling it exhibited slower time-dependent sensitivity indices.

The sensitivity analysis using Sobol’ first-order indices (Figs 2 and 3) revealed that all kinetic parameters affecting sodium and potassium conductances in the Hodgkin-Huxley model impact output variance. This was expected because the Hodgkin-Huxley model functions as a concerted system in which all transitions result in channel opening. In contrast, Markov models that include closed-closed transitions tend to exert less influence on the model’s output sensitivity [57].

After analyzing parameter sensitivity under voltage-clamp conditions, I integrated sodium and potassium conductances with the Hodgkin-Huxley model’s structural parameters in a 10 cm cable with a 0.5 mm diameter. A 70-ms square current pulse was applied at 0.4 cm from the sealed end to minimize end effects. For most simulations, I randomly sampled 300,000 parameter sets from Gaussian distributions based on the ranges listed in Table 1. Additionally, to ensure comprehensive coverage of the parameter space, I conducted simulations with 800,000 and 1,200,000 parameter sets, yielding results identical to those obtained with 300,000 sets (S3 Fig).

Fig 4A shows 500 individual simulations from this population, each receiving a 5 µA current injection through the electrode. The population average across all 300,000 simulations is shown in red and overlaid on these traces. The red trace is the pointwise average across all simulations and should not be interpreted as the response of a model with mean parameters. Because the system is nonlinear and the population contains multiple firing phenotypes, this population average is a descriptive summary of the ensemble rather than a representative individual trajectory.

**Fig 5:**
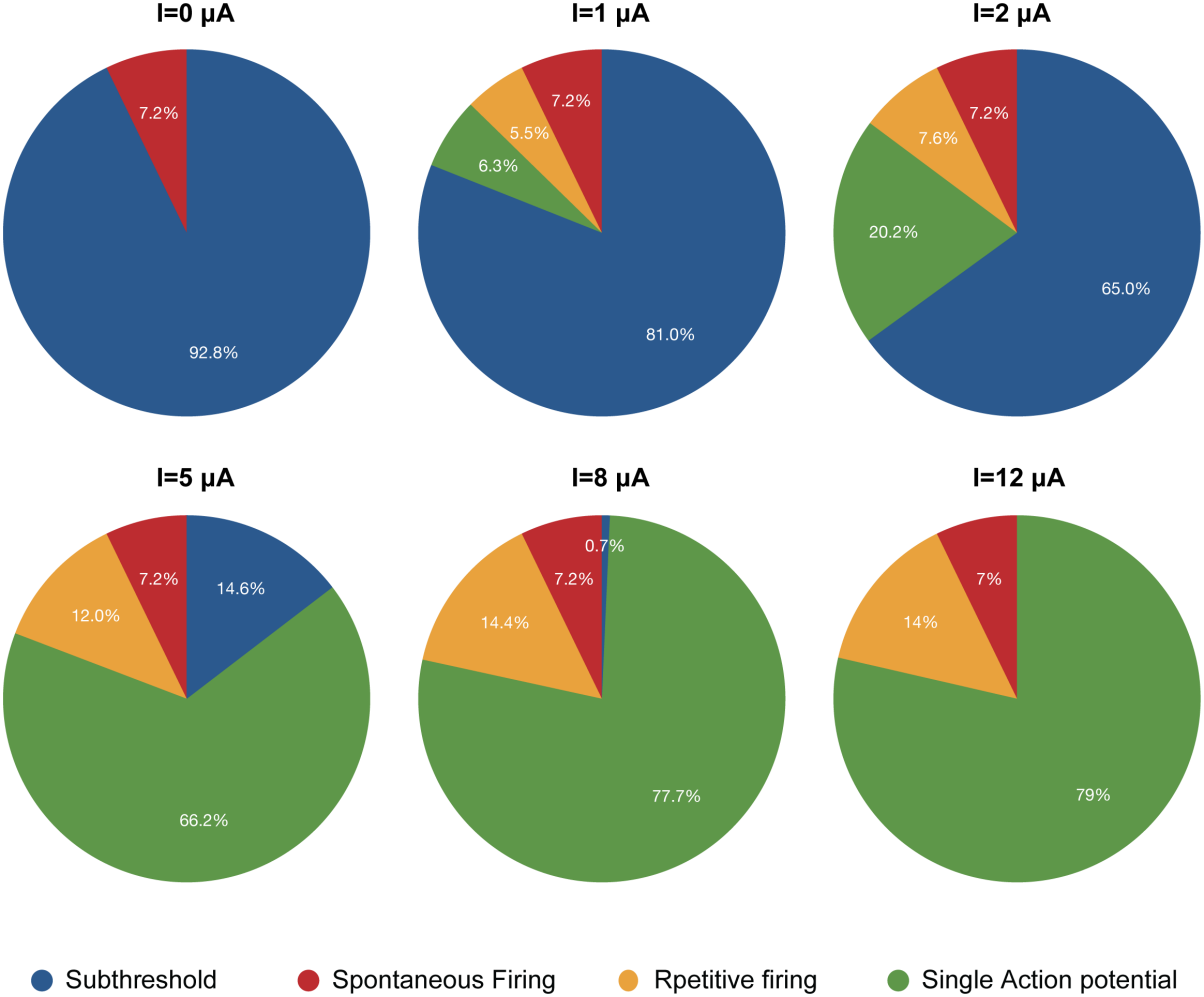
Population structure of firing behaviors in the Hodgkin-Huxley axon model as a function of injected current. Pie graphs show the relative numbers of each firing behavior observed in the Monte Carlo simulations. Subthreshold responses (blue), phasic responses that generate a single action potential at stimulus onset (green), repetitive firing responses that produce multiple action potentials during the stimulus (yellow), and spontaneous firing responses in which action potentials occur in the absence of injected current (red). The injected current is indicated above each pie graph.

**Fig 6:**
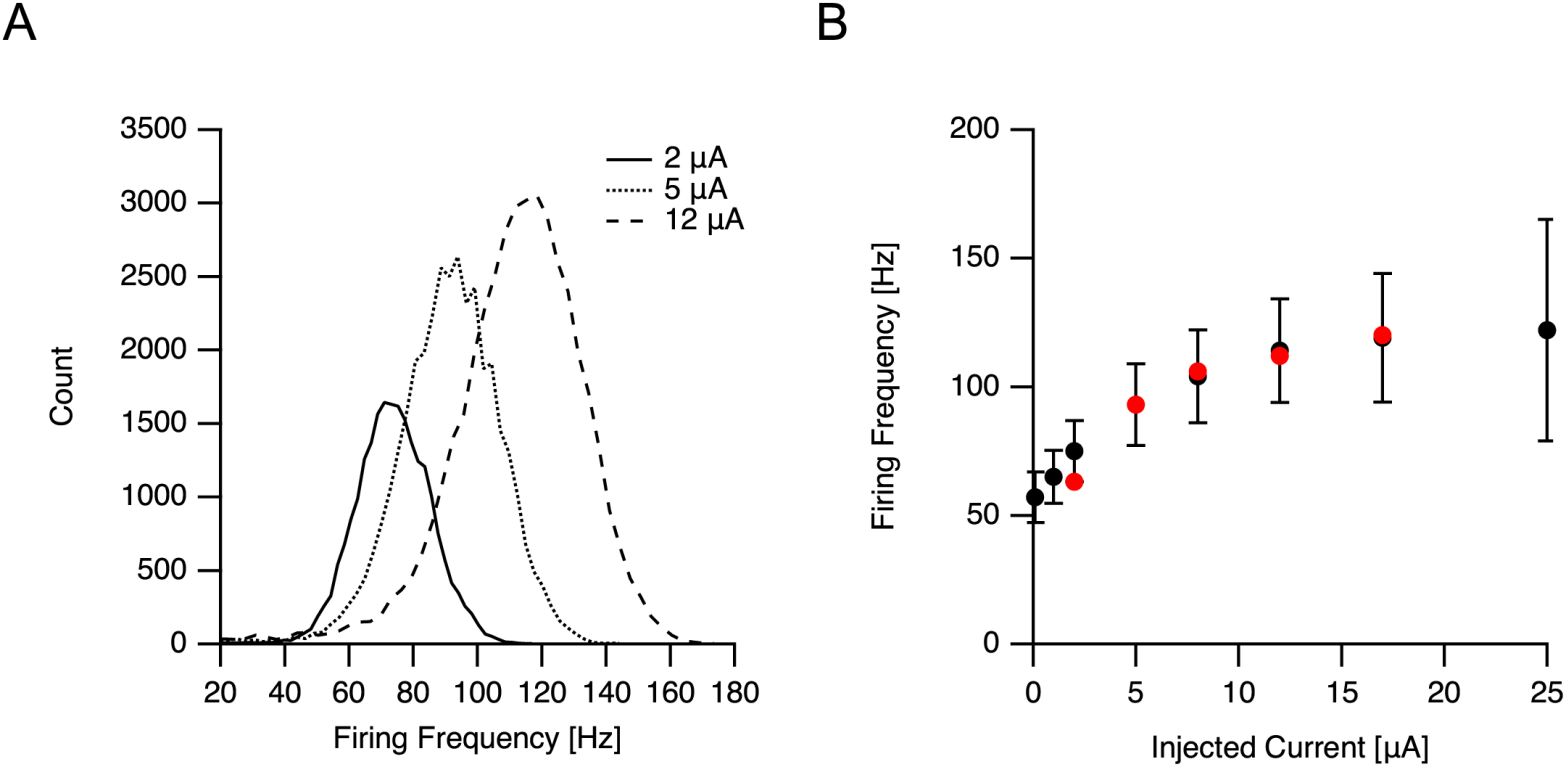
Firing rate variability in the regularly firing subpopulation. A, Histograms of firing rates for simulations classified as regularly firing, shown for three injected current amplitudes. Each histogram summarizes the distribution of firing frequencies across all parameter sets that produced sustained repetitive firing under the corresponding stimulus. The histograms were generated by current injections of 2 µA (lines), 5 µA (dots), and 12 µA (dashes). B, Mean firing rate (symbols) and standard deviation (error bars) of the regularly firing subpopulation plotted as a function of injected current amplitude. For comparison, the firing rates obtained from simulations using the original Hodgkin-Huxley parameter set are shown in red. The simulations using the original Hodgkin-Huxley parameter set were carried out in the same structural model and current injection levels as the rest of the simulations presented in this figure.

Based on their firing patterns, these traces were categorized into four subpopulations: traces in which no APs were generated at the injection site throughout the entire 100 ms simulation duration (Fig 4B), traces characterized by exactly one action potential occurring after stimulus onset (Fig 4C), traces showing two or more action potentials during the stimulus period (Fig 4D), and traces exhibiting one or more spontaneous action potentials during the initial 10 ms period before stimulus onset (Fig 4E). Action potentials were identified using an upward threshold-crossing method with a detection threshold of -20 mV. This automatic method for selecting action potentials was verified by manually inspecting 500 random traces from each subcategory. Overall, 7.2% of the simulations exhibited spontaneous firing (Fig 5). When no current was injected, the remaining population did not fire action potentials (Fig 5, top left corner). As the current increased, the proportion of phasic and regular firing subpopulations grew (Fig 5). The subthreshold response nearly disappeared as the current reached 8 µA. Interestingly, the regular-firing subpopulation increased to ~14%, whereas most simulations (77.7% at 8 µA) produced a phasic response. I then examined the axon subpopulation that fired repeatedly during stimulation. Fig 6A shows three firing rate histograms of this subpopulation at different current levels. Within the parameter ranges in my bootstrap analysis, each current injection produced a wide distribution of regular firing frequencies. Their mean and standard deviation are plotted in Fig 6B as a function of the injected current. For comparison, I simulated the original Hodgkin-Huxley model parameters. I calculated the firing rate as a function of current. As expected from the similarity between the mean parameters I extracted in the bootstrap analysis and the original Hodgkin-Huxley parameters (Table 1), these firing frequencies (red symbols in Fig 6B) matched the mean firing rate of the regularly firing subpopulation.

This result shows that the original Hodgkin-Huxley firing pattern is not the dominant behavior of the uncertainty-constrained ensemble. Rather, the canonical parameter set lies within the regularly firing subpopulation, whose mean firing-rate behavior it closely matches. Thus, the classical model remains representative of a single structured region of the parameter space, but it does not capture the full behavior of the experimentally constrained ensemble.

The predominance of the phasic response in the simulated population was unexpected, given that the Hodgkin-Huxley model typically generates a regular firing pattern. I anticipated that the subpopulation characterized by regular firing would be the largest. However, the phasic response better reflects the physiological role of the squid’s giant axon [58–61]. Consequently, the subsequent analyses focused on simulations where phasic firing produced a single action potential.

**Fig 7:**
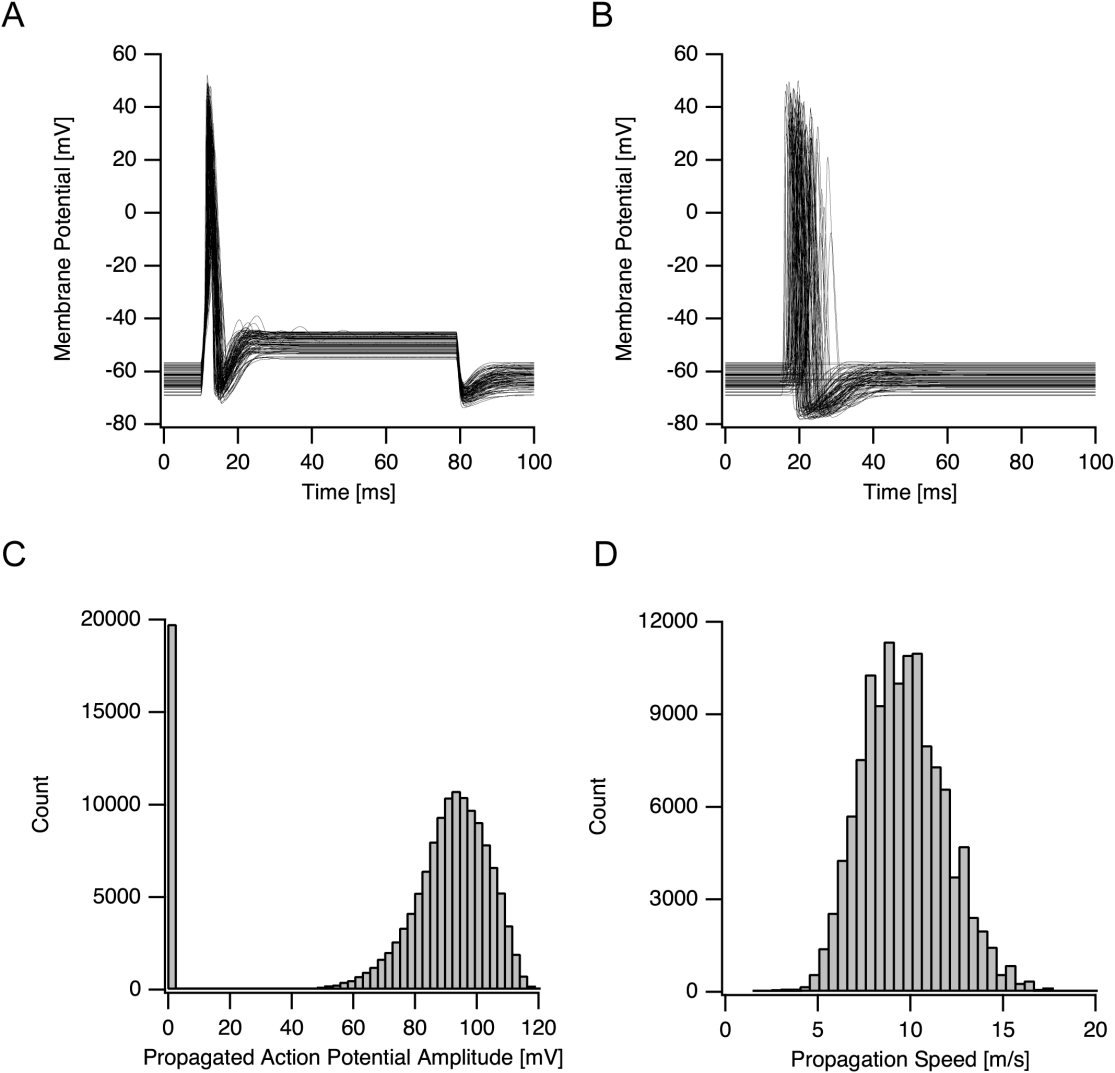
Propagation and variability of phasic action potentials in the axon model. A, Five hundred representative simulations from the phasic firing subpopulation, showing membrane potential traces recorded at the current injection site in response to a suprathreshold stimulus. B, Corresponding membrane potential traces recorded at a distal location 8 cm from the injection site, illustrating successful and failed propagation of action potentials along the cable. C, Distribution of peak action potential amplitudes at the distal recording site, reflecting variability in propagation efficacy, including complete propagation failures and attenuated spikes. D, Distribution of conduction velocities computed for successfully propagated action potentials.

I assessed whether the action potential propagated from its initiation site and measured its propagation speed. Fig 7A shows 500 overlaid simulations of phasic firing in response to a 5 µA injection at X = 0.4 cm. The propagated membrane potential at X = 8 cm is illustrated in Fig 7B. Successful propagation was defined by a distal action potential amplitude of 70 mV, while traces exhibiting subthreshold depolarization or decremental conduction below this threshold were classified as failed propagation. As expected, some action potentials successfully initiated at the current injection site but failed to reach the distal recording site, resulting in a broad amplitude distribution at the distal site (Fig 7C). These propagation outcomes provide an additional constraint that could be used to refine parameter ranges in future analyses. The conduction velocity was calculated by dividing the distance between the injection site and the distal recording site by the time delay between the action potential peaks at the two locations. The propagation speed in these simulations ranged from approximately 5–15 m/s (Fig 7D), consistent with experimental data [60,62].

**Fig 8:**
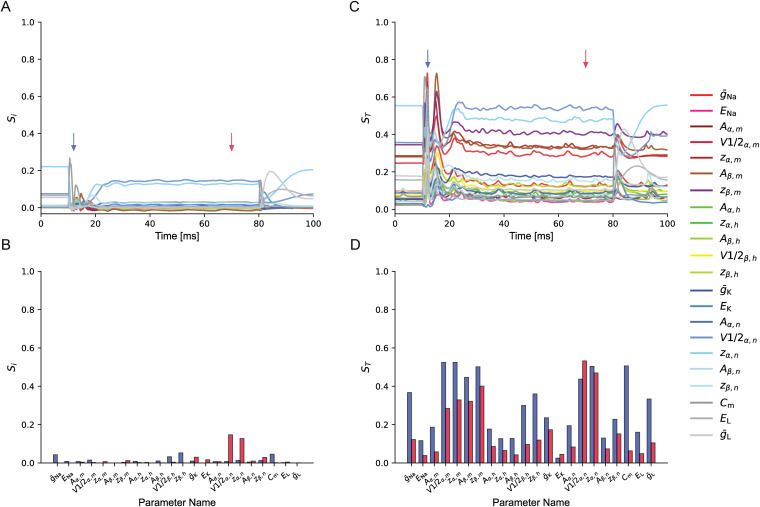
Global sensitivity structure of excitability in the phasic firing subpopulation. A, Time-dependent first-order Sobol sensitivity indices (S_i_) during the action potential and the subsequent sustained membrane potential, computed across simulations classified as phasic firing. B, Values of S_i_ during the action potential at the injection site (indicated by a blue arrow in A) and during the sustained membrane potential later in the simulation (indicated by a magenta arrow in **A)**. C, Time-dependent total-order Sobol sensitivity indices (ST_i_) during the action potential and the subsequent sustained membrane potential, computed across simulations classified as phasic firing. D, Values of ST_i_ during the action potential at the injection site (indicated by a blue arrow in C) and during the sustained membrane potential later in the simulation (indicated by a magenta arrow in **C)**. The simulation generated 376,832 traces (N = 8192 in Saltelli sampling for 22 parameters), out of which 182,386 displayed an action potential at stimulus onset. Sensitivity analysis presented in the figure was performed on this population.

I calculated the first- and total-order sensitivity indices (Fig 8) for the subpopulation of simulations exhibiting phasic firing (Fig 7A), as these may be of physiological importance. These simulations were carried out in a single-compartment Hodgkin-Huxley model to reduce computational load. To validate these simulations, I performed the same analyses shown in Figs 4-6 on the point neuron firing patterns, yielding similar results. The simulation generated 376,832 traces (N = 8192, Saltelli sampling for 22 parameters), out of which 182,386 displayed an action potential at stimulus onset. Sensitivity analysis presented in Fig 8 was performed on this population. As expected, the first-order sensitivity indices were small during both the action potential (at t = 12 ms, indicated by a blue arrow in Fig 8A and 8B) and the subsequent sustained membrane potential (at t = 70 ms, indicated by a magenta arrow in Fig 8A and 8B). The sustained potential was influenced mainly by two parameters of the potassium conductance (V1/2_α,n_ and A_β,n_), consistent with their role in the depolarization block following the initial firing of a single action potential (Fig 7A). The low first-order indices indicate that individual parameters had little independent effect after integration of the coupled differential equations. In contrast, the total-order sensitivity indices were high for nearly all parameters during both the initial action potential and the sustained membrane potential (Fig 8C and 8D), indicating strong interdependence among parameters due to their coupling with membrane potential.

## Discussion

Here, I show that neuronal excitability in the Hodgkin-Huxley framework is better understood as an ensemble property than as the output of a single parameter set. Re-fitting the original sodium and potassium rate-constant data by bootstrap resampling revealed substantial, parameter-specific uncertainty in the fitted equations (Fig 1 and Table 1). Propagating this uncertainty through an excitable cable model produced a heterogeneous population of firing behaviors, non-firing, phasic, regular, and spontaneous, in which the dominant response across stimulus amplitudes was phasic firing (Figs 4 and 5). The canonical Hodgkin–Huxley parameter set itself fell within a regularly firing minority whose mean firing rate it closely matched (Fig 6), and in the phasic subpopulation, action-potential propagation remained within experimentally realistic conduction velocities (Fig 7). Global sensitivity analysis during spiking showed small first-order Sobol indices but large total-order indices (Fig 8), indicating that excitability depends primarily on interactions among parameters rather than on any single kinetic or conductance parameter.

A key result is that parameter sensitivity depends strongly on the level of description at which the model is interrogated. Under voltage-clamp conditions, individual kinetic parameters exert pronounced, time-dependent first-order effects on channel behavior (Figs 2 and 3). In this regime, sensitivity indices reflect the relatively direct control of specific rate constants over activation and inactivation processes, closely mirroring the structure of the underlying equations. However, when these conductances are embedded in an excitable membrane and driven to spike, the situation changes qualitatively. During action potential generation, first-order Sobol indices were small (Fig 8A, 8B), indicating that individual parameters are not unimportant, but that their effects are not expressed independently in the integrated spiking system. At the same time, total-order indices remained large for nearly all parameters (Fig 8C and 8D), indicating that these parameters contribute substantially to output variance through interactions with other parameters. This distinction is central. Low first-order sensitivity does not imply weak influence; rather, it indicates that a parameter’s influence is conditional on the values of other parameters. In the Hodgkin-Huxley model, this coupling is mediated by the membrane potential: a change in one kinetic, conductance, or passive parameter alters the voltage, which in turn changes all voltage-dependent rates, which then feed back onto the voltage. The transition from isolated channel dynamics to integrated excitability, therefore, transforms parameter influence from relatively direct control into interaction-dominated control.

The two time points at which Fig 8 reports first- and total-order indices are not arbitrary. The index values during the action potential, at t = 12 ms, reflect the parameters that govern spike initiation, peak voltage, and the rising and falling phases of the spike, while the values during the sustained subthreshold potential, at t = 70 ms, reflect the parameters that govern the depolarization block and the post-spike membrane state. Together, they capture the main spike-shape and spike-timing features within a single Sobol decomposition. The qualitative pattern, with small first-order indices and large total-order indices, held at both time points and across all 22 parameters, indicating that interaction-dominated sensitivity is a robust feature of integrated excitability rather than a property of any one spike feature. Where a small number of parameters exerted a non-negligible first-order influence on the sustained potential, namely V_1/2α,n_ and A_β,n_ of the potassium conductance, this is consistent with their direct role in the depolarization block following the initial spike. This provides a useful example of how channel-level sensitivity structure can surface in the integrated system at specific phases of the response.

The population simulations further demonstrate that preserving parameter uncertainty fundamentally alters the model’s qualitative behavior. Sampling hundreds of thousands of parameter sets produced a heterogeneous ensemble of responses, including non-firing, phasic firing, regular firing, and spontaneous activity (Figs 4 and 5). Notably, the dominant subpopulation consisted of models that fired a single action potential in response to sustained depolarization (Fig 4C), while regularly firing models constituted a minority. At first glance, this result appears to contradict the behavior of the original Hodgkin-Huxley model, which typically produces repetitive firing under constant current injection. However, when interpreted in light of the squid giant axon’s physiological role, the predominance of phasic firing emerges as a biologically appropriate outcome rather than an anomaly.

The giant axon of the squid is a highly specialized structure that triggers rapid, all-or-none contraction of the mantle during escape behavior [58,59]. Experimental studies have shown that, under natural conditions, the axon typically fires a single action potential in response to sensory stimuli, particularly visual flashes in cold seawater, initiating a powerful jet-propelled response [59–64]. More complex patterns, one to three spikes or even complete silence, occur only in delayed escape behaviors involving chemical stimulation and coordination with the parallel small-axon system. This firing behavior reflects the axon’s intrinsic excitability, in which a neuron responds to sustained depolarization with a single spike followed by silence [65,66]. The predominance of phasic firing in the simulations, therefore, closely aligns with the known physiological function of the squid giant axon. This alignment suggests that the classic Hodgkin-Huxley parameter set represents one region of a broader, physiologically relevant parameter landscape rather than a uniquely privileged description of the axon’s functional behavior.

These findings extend the population view established by previous work on conductance degeneracy. The database approach to biophysical modeling has traditionally focused on the variability of conductance densities while holding channel kinetics constant. The important work by Prinz et al. [19] demonstrated that similar network activity can arise from disparate sets of maximal conductances, establishing the concept of multiple solutions in neuronal modeling. Subsequent studies [17,28,67–70] expanded this framework to include morphological variability and other sources of cellular diversity. Automated optimization studies reached a related practical conclusion: genetic algorithms and multi-objective optimization can identify many conductance-based models that reproduce selected experimental features [22,23,34,35,39–41,43,71]. In most of these workflows, however, voltage-dependent rate constants are treated as a fixed scaffold on which conductance densities and other parameters are tuned. The present study extends the same population logic to the kinetic parameters themselves. This distinction matters because maximal conductances act primarily as scaling factors, whereas kinetic parameters determine the timing and nonlinear state transitions that shape spike initiation, refractoriness, and propagation. The global sensitivity analysis presented here shows that kinetic parameters, often frozen in database exploration and standard optimization pipelines, remain consequential in the integrated system through higher-order interactions with conductance and passive parameters (Fig 8). This supports the observations of Goaillard et al. [29], who noted that ion channel degeneracy involves covariation in biophysical properties beyond simple conductance magnitudes. Modeling robustness solely through conductance variation, therefore, overlooks a substantial dimension of model variability and may underestimate the compensatory mechanisms available to excitable systems [72].

Ori et al. provide an elegant and highly instructive demonstration that, despite the apparent high dimensionality of the Hodgkin-Huxley model, the functional outcome of excitability can be organized in a much lower-dimensional physiological space [27]. Conceptually, this work anticipated the need to move beyond single parameter sets and to view excitability as a population-level property of the Hodgkin-Huxley model. In their analysis, they systematically rescaled the original Hodgkin-Huxley rate constants across a broad range of independent values. They showed that the resulting behaviors cluster along two composite axes that capture structural and kinetic contributions. They propose that slow sodium inactivation is a local homeostatic mechanism that stabilizes excitability within this reduced plane. In the present manuscript, I approached the same overarching question from a complementary direction, by grounding parameter dispersion in a data-driven uncertainty analysis of the original voltage-clamp figures and using bootstrapping to estimate parameter-specific variability rather than imposing uniform rescaling across rate constants. While Ori et al. isolate a powerful geometric principle and mechanistic stabilizer using short-pulse stimulation in a single-compartment setting, the present results emphasize how experimentally constrained, parameter-specific uncertainty and parameter interactions shape spike initiation, waveform, and propagation in an extended axon, and why kinetic parameters must be treated on equal footing with conductances when interpreting robustness and degeneracy in Hodgkin-Huxley-type models.

A complementary line of work has approached the same broad question through simulation-based inference, which uses likelihood-free Bayesian inference and neural density estimation to recover posterior distributions over biophysical parameters [31,32]. As mentioned in the Introduction, at the channel level, simulation-based inference can recover joint posteriors over the parameters that define steady-state activation and time-constant curves, including non-trivial covariance structure between kinetic parameters. At the level of integrated Hodgkin-Huxley-type spiking models, however, simulation-based inference applications typically inherit channel kinetics as a prescribed model structure [33, 73] and infer over conductances and a small set of additional membrane or adaptation parameters. The present study addresses a level of the modeling pipeline that has received less direct attention in these approaches: the uncertainty introduced when channel rate constants are fit from experimental voltage-clamp data and then frozen before incorporation into a spiking model. The complementarity between the two approaches is methodological rather than competitive. Bootstrap-derived parameter distributions and simulation-based inference posteriors are different objects: the former quantify how reproducibly rate-constant fits can be recovered from resamples of the original measurements, while the latter quantify which parameters are consistent with chosen summary features of the data under a chosen model and prior. The two can, in principle, be combined, with bootstrap distributions providing data-driven priors for spiking-level simulation-based inference.

This complementarity raises a concrete methodological question that is beyond the scope of the present manuscript but follows directly from its findings. The standard practice of fitting conductance-based models under a fixed-kinetics assumption, whether by genetic algorithms, multi-objective optimization, or simulation-based inference conditioned on a small feature set, may bias estimates of maximal conductances whenever the underlying kinetics differ from the prescribed kinetic model. The present results identify the precondition for such bias: kinetic parameters shape excitability through interactions with conductance and passive parameters (Fig 8). An optimizer may therefore compensate for deviations in kinetics by adjusting gNa and gK along directions in parameter space that preserve the targeted firing features. This suggests a testable prediction: fitting conductances under fixed canonical kinetics to data generated from a broader kinetic distribution should produce conductance estimates whose bias depends on the deviation of the true kinetics from the canonical values. Testing this prediction would require a dedicated inference framework and target feature set, and is therefore a separate study. The point here is to make explicit a methodological consequence of the present analysis for the construction and evaluation of detailed conductance-based models.

Despite this convergence with physiology, the variability extracted in this study has clear limitations. Parameter uncertainty was estimated after selecting a specific kinetic model and was based on digitized data rather than original voltage-clamp recordings. As a result, the inferred uncertainty should not be interpreted as pure biological variability. It reflects a mixture of experimental scatter, digitization error, fitting uncertainty, assumptions imposed by the chosen Hodgkin-Huxley functional forms, and biological variability. The analysis also does not include model-form uncertainty: alternative kinetic descriptions, such as Markov models or modified Hodgkin-Huxley rate equations, could yield different uncertainty structures and sensitivities. In addition, the Monte Carlo sampling assumed independent variation of parameters, even though fitted kinetic parameters may covary, as amplitudes, voltage offsets, slope factors, and forward and backward rates can compensate for one another during curve fitting. Finally, the spatial model used a fixed cable morphology and fixed compartmental structure, and therefore did not include variability in axon diameter, morphology, or spatial channel distributions, all of which can influence excitability and conduction velocity. These limitations define the scope of the present analysis rather than invalidate its conclusions. Even under this constrained reconstruction, a large region of parameter space produced phasic firing and realistic propagation behavior, including conduction velocities within experimentally observed ranges (Fig 7D).

Not all parameters exhibited the same level of uncertainty. The bootstrap analysis revealed substantial heterogeneity in parameter variance (Table 1), reflecting differences in data density, voltage coverage, and scatter in the original Hodgkin-Huxley figures. Within each rate constant, parameters also trade off against one another, since the same data can be fitted equally well by adjusting amplitudes, voltage offsets, or slope factors, or by balancing forward and backward rates. Consistent with this, the bootstrap fits show structured pairwise correlations within rate constants and negligible correlations across rate constants (S1 and S2 Figs). This redundancy is a property of the fitting geometry, not of channel function: many different parameter combinations produce nearly indistinguishable activation and inactivation curves.

The Monte Carlo analysis was deliberately performed without imposing these within-rate-constant correlations on the sampling, partly to test whether structured firing behavior would emerge despite their absence. That it did suggests that the population structure described here is not an artifact of correlated sampling, and that incorporating empirical correlations into future Monte Carlo analyses would refine, rather than overturn, the present findings. The broader implication is that even when correlations reduce the apparent dimensionality of the kinetic parameter space, the underlying degrees of freedom remain consequential for spiking: individual parameters have weak independent effects in the integrated system, but their combined interactions strongly shape excitability (Fig 8). This is precisely why sensitivity analysis must be performed on the coupled system rather than on isolated parameters.

More broadly, the results presented here point toward an alternative workflow for biophysical modeling. Rather than seeking a single optimized parameter set, the findings argue for a process in which uncertainty analysis is performed first, followed by generation of a large ensemble of models that is analyzed as a structured population. Within such populations, physiological behavior corresponds to regions of parameter space rather than isolated points. Selecting a single “best” model, whether by manual tuning or automated optimization, inevitably discards this structure and is somewhat analogous to overfitting. Even after constraining models to match physiological behavior, a nontrivial parameter manifold remains, and simulations must therefore account for variability rather than collapsing it into an average.

Finally, the present work also highlights directions for future extension. The analysis did not include variability in axon diameter or morphology, factors known to influence excitability and conduction velocity strongly. Incorporating morphological uncertainty, additional conductances, or alternative channel formalisms such as Markov models will further enrich population-level analyses. Markov models, in particular, allow description of more complex kinetics than the Hodgkin-Huxley formalism, but also introduce transitions that may contribute weakly to functional sensitivity [57]. Applying the same uncertainty- and interaction-focused framework to such models will help clarify which aspects of added biophysical detail meaningfully shape neuronal behavior.

In summary, the Hodgkin-Huxley model remains a uniquely powerful foundation for understanding neuronal excitability, and the present analysis does not attempt to challenge that status. What it does show is that, when experimentally grounded uncertainty in the fitted rate parameters is preserved and propagated, the model produces a structured population of firing behaviors dominated by phasic responses, consistent with the physiological role of the squid giant axon, within which the classical 1952 parameter set sits as a regularly firing minority. Excitability in this population is governed by interactions among kinetic, conductance, and passive parameters rather than by any single one. The Hodgkin-Huxley model is therefore best understood not as a single privileged parameter set, but as an experimentally constrained ensemble of behaviors.

## Supporting information

S1 FigPairwise bootstrap distributions of potassium conductance (n-gate) parameters.Each panel shows the joint distribution of two parameters obtained from ~4,000 bootstrap replicates of the potassium channel n-gate kinetics. Diagonal panels display the marginal distribution of each parameter as a histogram. Off-diagonal panels display pairwise scatter plots of the corresponding bootstrap samples. Color indicates the rate constant to which both parameters belong: blue for the forward rate constant $\alpha_{n}$ ($A_{\alpha ,n}$, ${V\text{1}/\text{2}}_{\alpha ,n}$, $z_{\alpha ,n}$) and green for the backward rate constant $\beta_{n}$ ($A_{\beta ,n}$, $z_{\beta ,n}$). Panels comparing parameters from different rate constants are shown in black. Because $\alpha_{n}$ and $\beta_{n}$ were fitted independently, cross-rate-constant panels (black) show no appreciable correlation, whereas within-rate-constant panels (colored) reflect the parameter trade-offs imposed by the data within each rate equation.(PDF)

S2 FigPairwise bootstrap distributions of sodium conductance parameters.Each panel shows the joint distribution of two parameters obtained from ~4,000 bootstrap replicates of the sodium channel kinetics, spanning all four rate constants governing activation ($\alpha_{m}$, $\beta_{m}$) and inactivation ($\alpha_{h}$, $\beta_{h}$). Diagonal panels display the marginal distribution of each parameter as a histogram. Off-diagonal panels display pairwise scatter plots of the corresponding bootstrap samples. Color indicates the rate constant to which both parameters belong: red for $\alpha_{m}$ ($A_{\alpha ,m}$, ${V\text{1}/\text{2}}_{\alpha ,m}$, $z_{\alpha ,m}$), purple for $\beta_{m}$ ($A_{\beta ,m}$, $z_{\beta ,m}$), cyan for $\alpha_{h}$ ($A_{\alpha ,h}$, $z_{\alpha ,h}$), and orange for $\beta_{h}$ ($A_{\beta ,h}$, ${V\text{1}/\text{2}}_{\beta ,h}$, $z_{\beta ,h}$). Panels comparing parameters from different rate constants are shown in black. As in S1 Fig, correlations are concentrated within individual rate constants (colored panels) and are negligible across rate constants (black panels), consistent with the independent bootstrap fitting of each rate equation. The asymmetric distribution of $A_{\beta ,m}$ (purple diagonal panel) reflects the limited number of data points and large scatter in the $\beta_{m}$ measurements.(PDF)

S3 Fig Testing Monte Carlo sample size. Distribution of the four firing subcategories obtained when the simulation was performed with a population of 300,000, 800,000, and 1,200,000 parameter sets. The contribution of each subcategory to the population was identical across these simulations.
(PDF)
