# Supplementary material for: Neuronal excitability and parameter variability in the Hodgkin-Huxley model
Source: PLoS Comput Biol. 2026 Jun 29;22(6):e1014458. doi: 10.1371/journal.pcbi.1014458 (PMC13336477; doi:10.1371/journal.pcbi.1014458)
Supplement: S1 Fig — Each panel shows the joint distribution of two parameters obtained from ~4,000 bootstrap replicates of the potassium channel n-gate kinetics. Diagonal panels display the marginal distribution of each parameter as a histogram. Off-diagonal panels display pairwise scatter plots of the corresponding bootstrap samples. Color indicates the rate constant to which both parameters belong: blue for the forward rate constant αn (Aα,n, V1/2α,n, zα,n) and green for the backward rate constant βn (Aβ,n, zβ,n). Panels comparing parameters from different rate constants are shown in black. Because αn and βn were fitted independently, cross-rate-constant panels (black) show no appreciable correlation, whereas within-rate-constant panels (colored) reflect the parameter trade-offs imposed by the data within each rate equation. (PDF) [file pcbi.1014458.s001.pdf]

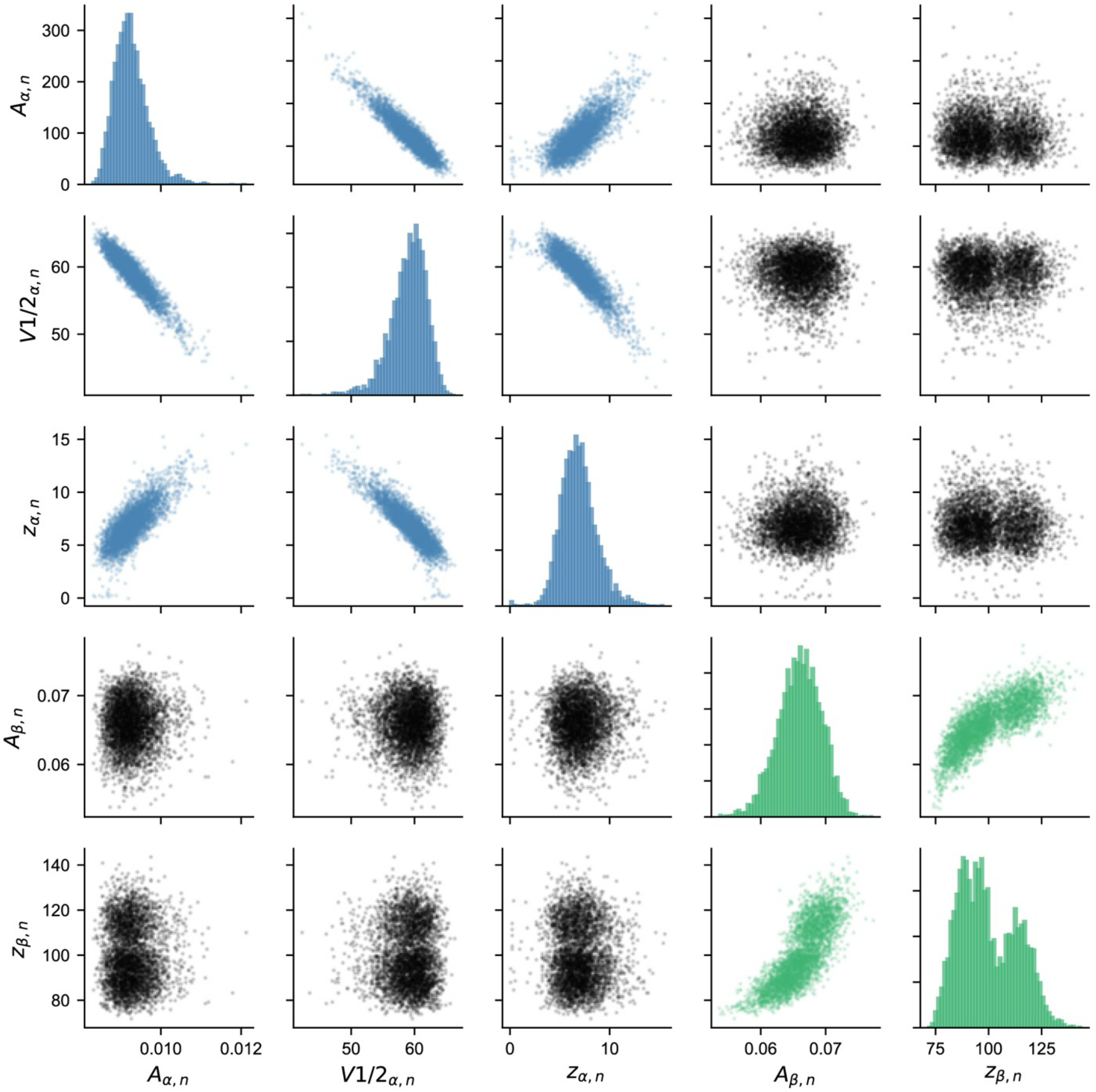

Supplementary Figure 1. Pairwise bootstrap distributions of potassium conductance (n-gate) parameters.

Each panel shows the joint distribution of two parameters obtained from ~4,000 bootstrap replicates of the potassium channel n-gate kinetics. Diagonal panels display the marginal distribution of each parameter as a histogram. Off-diagonal panels display pairwise scatter plots of the corresponding bootstrap samples. Color indicates the rate constant to which both parameters belong: blue for the forward rate constant α_n ($A_{\alpha,n}, V1/2_{\alpha,n}, z_{\alpha,n}$) and green for the backward rate constant β_n ($A_{\beta,n}$, $z_{\beta,n}$). Panels comparing parameters from different rate constants are shown in black. Because α_n and β_n were fitted independently, cross-rate-constant panels (black) show no appreciable correlation, whereas within-rate-constant panels (colored) reflect the parameter trade-offs imposed by the data within each rate equation.
