# Supplementary material for: Neuronal excitability and parameter variability in the Hodgkin-Huxley model
Source: PLoS Comput Biol. 2026 Jun 29;22(6):e1014458. doi: 10.1371/journal.pcbi.1014458 (PMC13336477; doi:10.1371/journal.pcbi.1014458)
Supplement: S2 Fig — Each panel shows the joint distribution of two parameters obtained from ~4,000 bootstrap replicates of the sodium channel kinetics, spanning all four rate constants governing activation (αm, βm) and inactivation (αh, βh). Diagonal panels display the marginal distribution of each parameter as a histogram. Off-diagonal panels display pairwise scatter plots of the corresponding bootstrap samples. Color indicates the rate constant to which both parameters belong: red for αm (Aα,m, V1/2α,m, zα,m), purple for βm (Aβ,m, zβ,m), cyan for αh (Aα,h, zα,h), and orange for βh (Aβ,h, V1/2β,h, zβ,h). Panels comparing parameters from different rate constants are shown in black. As in S1 Fig, correlations are concentrated within individual rate constants (colored panels) and are negligible across rate constants (black panels), consistent with the independent bootstrap fitting of each rate equation. The asymmetric distribution of Aβ,m (purple diagonal panel) reflects the limited number of data points and large scatter in the βm measurements. (PDF) [file pcbi.1014458.s002.pdf]

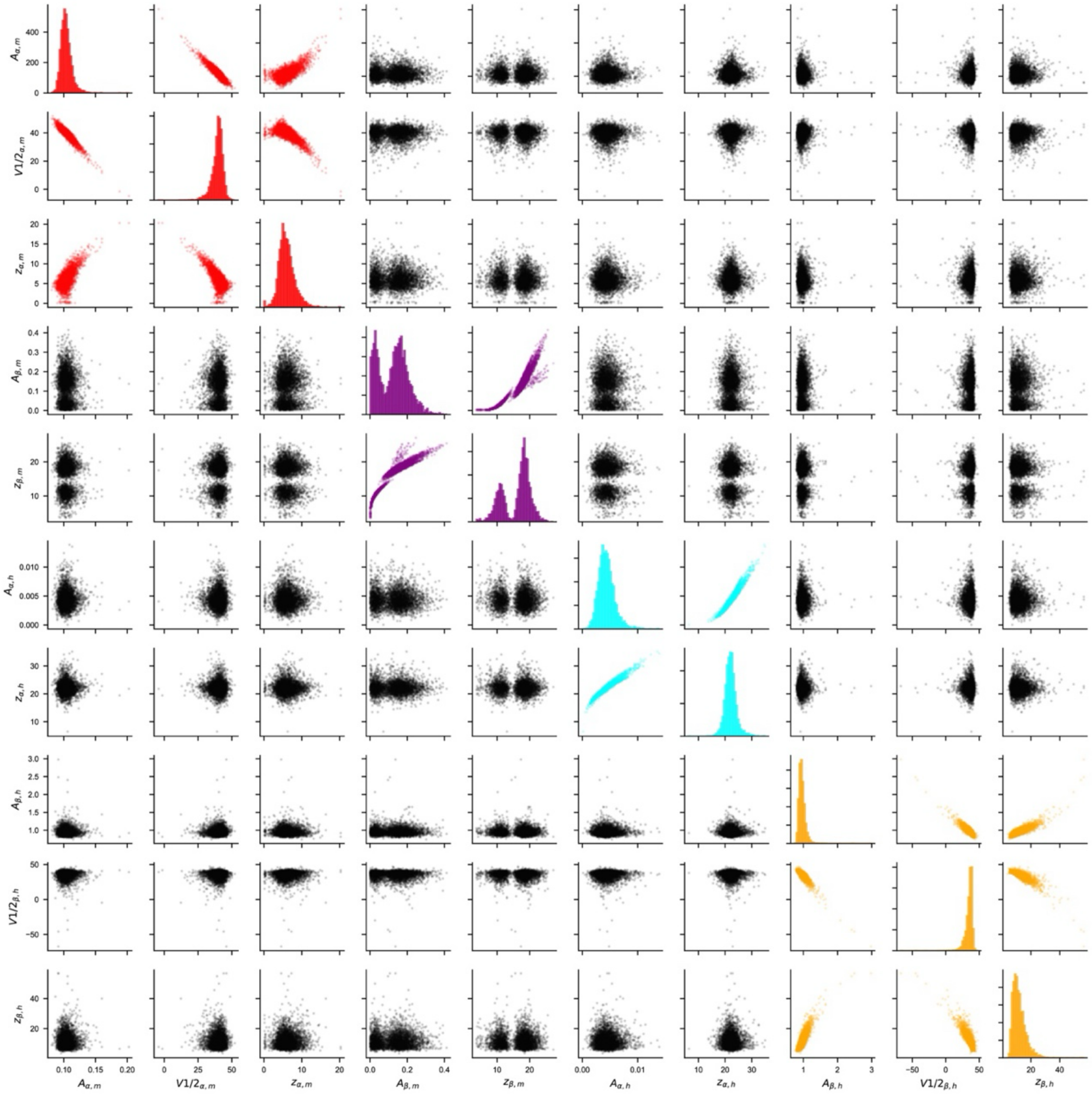

Supplementary Figure 2. Pairwise bootstrap distributions of sodium conductance parameters.

Each panel shows the joint distribution of two parameters obtained from ~4,000 bootstrap replicates of the sodium channel kinetics, spanning all four rate constants governing activation (α_m, β_m) and inactivation (α_h, β_h). Diagonal panels display the marginal distribution of each parameter as a histogram. Off-diagonal panels display pairwise scatter plots of the corresponding bootstrap samples. Color indicates the rate constant to which both parameters belong: red for α_m ($A_{\alpha,m}, V1/2_{\alpha,m}, z_{\alpha,m}$), purple for β_m ($A_{\beta,m}, z_{\beta,m}$), cyan for α_h ($A_{\alpha,h}$, $z_{\alpha,h}$), and orange for β_h ($A_{\beta,h}$, $V1/2_{\beta,h}$, $z_{\beta,h}$). Panels comparing parameters from different rate constants are shown in black. As in Supplementary Figure 1, correlations are concentrated within individual rate constants (colored panels) and are negligible across rate constants (black panels), consistent with the independent bootstrap fitting of each rate equation. The asymmetric distribution of $A_{\beta,m}$ (purple diagonal panel) reflects the limited number of data points and large scatter in the β_m measurements.
