# Supplementary material for: Neuronal excitability and parameter variability in the Hodgkin-Huxley model
Source: PLoS Comput Biol. 2026 Jun 29;22(6):e1014458. doi: 10.1371/journal.pcbi.1014458 (PMC13336477; doi:10.1371/journal.pcbi.1014458)
Supplement: S3 Fig — Distribution of the four firing subcategories obtained when the simulation was performed with a population of 300,000, 800,000, and 1,200,000 parameter sets. The contribution of each subcategory to the population was identical across these simulations. (PDF) [file pcbi.1014458.s003.pdf]

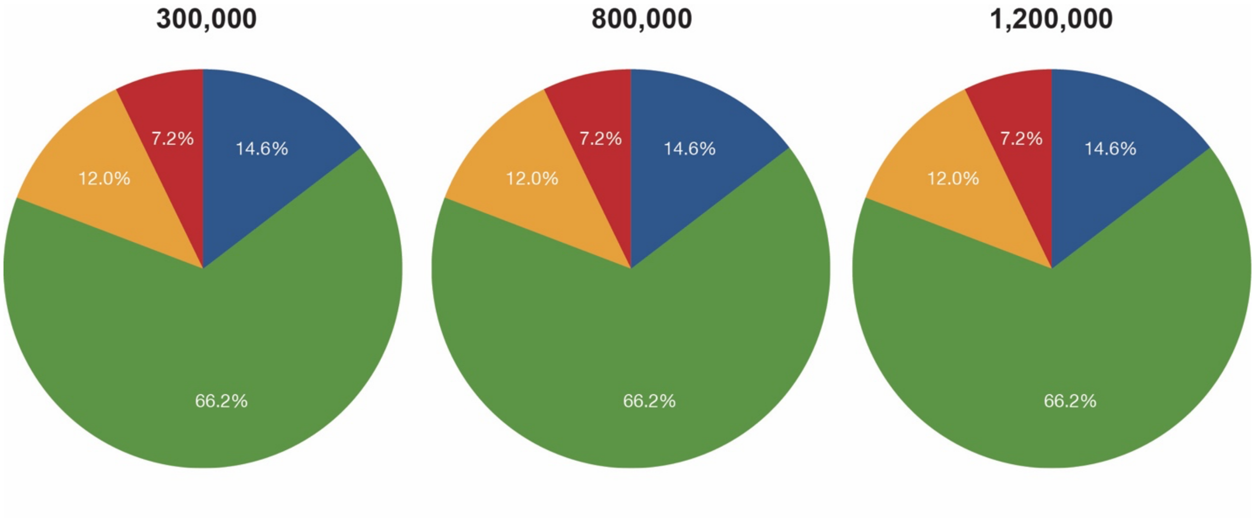

Supplementary Figure 3: Testing Monte Carlo sample size. Distribution of the four firing subcategories obtained when the simulation was performed with a population of 300,000, 800,000, and 1,200,000 parameter sets. The contribution of each subcategory to the population was identical across these simulations.
