# Supplementary material for: Neuronal excitability and parameter variability in the Hodgkin-Huxley model
Source: PLoS Comput Biol. 2026 Jun 29;22(6):e1014458. doi: 10.1371/journal.pcbi.1014458 (PMC13336477; doi:10.1371/journal.pcbi.1014458)
Supplement: Attachment — Submitted filename: reply5.pdf [file pcbi.1014458.s005.pdf]

I thank both reviewers for the careful and constructive reading of the manuscript. The reviews converged on two structural concerns that I have taken seriously and that, between them, have substantially reshaped the paper: that the framing did not foreground the specific contribution sharply enough against existing work on parameter degeneracy and simulation-based inference, and that the implications of the analysis were stated too narrowly within the Discussion and not pulled forward into the Introduction, Results, Abstract, or Author Summary. Together with several specific clarifications requested by each reviewer, these shared concerns motivated a more focused reframing of the central claim. The Hodgkin–Huxley model is now presented throughout the manuscript as an experimentally constrained ensemble of behaviors rather than as a single privileged parameter set, with biologically relevant function emerging from structured regions of parameter space. The Introduction, Abstract, Author Summary, Results, and Discussion have each been revised to state this reframing explicitly at the level appropriate to that section, and the specific contributions that distinguish the present work from prior population-based and inference-based studies, data-driven uncertainty estimation from the original Hodgkin–Huxley figures, the resulting phasic-dominant population structure, and the interaction-dominated Sobol decomposition during spiking, are now stated in the Introduction, previewed at the end of it, and reinforced in the Discussion.

Reviewer #1:

1. Novelty framing and engagement with simulation-based inference literature.
The paper cites much of the relevant prior work on parameter degeneracy and robustness (Prinz, Marder, Golowasch, Goldman, etc.), but the abstract and introduction sometimes read as though the core insight, that varying HH parameters produces heterogeneous firing behaviors and that parameter interactions matter, is itself novel. Since this is well established, the framing would benefit from more clearly foregrounding what this study specifically adds: the data-driven uncertainty estimation from the original HH figures, the resulting population structure (especially phasic firing dominance), and the formal Sobol decomposition. The contributions are genuine; they just need to be distinguished more from prior work. One concrete example: the Discussion claims that kinetic parameter variability has been largely unexplored and that variability analysis is typically performed post-hoc. However, work on simulation-based inference (SBI), notably Gonçalves et al. (2020, "Training deep neural density estimators to identify mechanistic models of neural dynamics," eLife), directly investigates variability and covariance across HH parameters, including kinetic parameters (see their Figure 3), and does so as part of the inference process rather than post-hoc. Acknowledging this work and related SBI literature would help position the present study's bootstrap-based approach more accurately within the field.

Reply: The Introduction and Discussion have been substantially restructured to foreground the specific contribution of this study and to position it accurately against the simulation-based inference literature.
In the Introduction, a new paragraph engages directly with Gonçalves et al. 2020 and Lueckmann et al. 2017 (now references [31] and [32]), acknowledging that simulation-based inference recovers posterior distributions over biophysical parameters and that, at the channel level, Gonçalves et al. infer parameters that define steady-state activation and time-constant curves with non-trivial covariance structure (their Figure 3). The Introduction then distinguishes where this literature has gone and where the present study fits: at the

level of integrated Hodgkin–Huxley spiking models, even within the SBI framework, channel kinetics are typically inherited as a prescribed model structure (now citing Pospischil et al. 2008 [33] and Destexhe & Huguenard 2000 [73]) while inference is performed over conductances and a small number of additional membrane or adaptation parameters. The framing has been softened from "largely unexplored" to "underexploited" and is now explicitly localized to the construction and analysis stages of the modeling pipeline.
A subsequent paragraph names the specific contribution in three points: bootstrap-based, data-driven uncertainty estimation from the original Hodgkin–Huxley figures; the resulting population structure (in particular the phasic-firing dominance and the position of the canonical 1952 parameter set within the regularly firing minority); and the formal Sobol decomposition that separates first- from total-order contributions during spiking. The final Introduction paragraph previews these three results explicitly.
In the Discussion, a new paragraph engages with the same SBI literature in light of the results. It frames the relationship between bootstrap-derived parameter distributions and simulation-based inference posteriors as methodologically complementary rather than competitive (the former quantify reproducibility of rate-constant fits from resamples of the original measurements; the latter quantify which parameters are consistent with chosen summary features under a chosen model and prior), and notes that the two approaches can in principle be combined, with bootstrap distributions providing data-driven priors for spiking-level simulation-based inference. I hope that, together with the new Discussion paragraph on the testable hypothesis for fixed-vs-free kinetics inference, this places the present bootstrap-based approach explicitly within the SBI literature rather than alongside it.

2. Bootstrap correlation structure.
Parameters are sampled independently from Gaussian distributions, but the authors note in the Discussion that rate-constant parameters are likely correlated. It would be valuable to report the correlation matrix from the bootstrap fits and briefly discuss which parameter pairs show strong correlations. This does not require additional simulations. The independent sampling is a reasonable choice, and the fact that structured results emerge from it is itself informative. But reporting the correlations would help readers assess the geometry of the parameter space and could motivate future work with correlated sampling.

Reply: I agree. The bootstrap correlation structure is now reported in two new supplementary figures and described in the Methods, Results, and Discussion. Specifically:
In the Methods, a new "Bootstrap parameter estimation" subsection documents the procedure (4,000 resamples per rate constant, independent fitting across rate constants) and notes that, because each rate constant was fitted separately, correlations among parameters reflect the local geometry of each rate equation, while parameters from different rate constants are largely uncorrelated.
In the Supplementary Information, two new figures replace the previous single marginal-distribution figure. Supplementary Figure 1 shows pairwise bootstrap distributions for the five potassium n-gate parameters; Supplementary Figure 2 shows pairwise bootstrap distributions for the ten sodium-channel parameters spanning αm, βm, αh, and βh. Diagonal panels show the marginal distributions and off-diagonal panels show pairwise

scatter plots, color-coded by which rate constant the two parameters belong to. This makes the geometry of the parameter space visible at a glance: correlations are concentrated within individual rate constants (colored panels) and are negligible across rate constants (black panels). The asymmetric distribution of $A_{\beta,m}$ is identified and attributed to the limited data and large scatter in the β_m measurements of the original Hodgkin-Huxley paper. The previous Supplementary Figure 2 (Monte Carlo sample-size convergence) has been renumbered to Supplementary Figure 3.
In the Results, the text now states that parameter correlations are concentrated within individual rate constants and points to the new figures.
In the Discussion, the correlations paragraph now points to Supplementary Figures 1 and 2 and makes explicit that the independent Monte Carlo sampling was a deliberate choice. The paragraph argues that because the structured firing population emerges despite the absence of imposed correlations, the result is not an artifact of correlated sampling, and that incorporating empirical correlations in future Monte Carlo analyses would be expected to refine, rather than overturn, the present findings, which may motivate future work.

3. Abstract and Methods clarity.
The abstract refers to "propagating uncertainty estimates through a spatially extended squid axon cable model using large-scale Monte Carlo simulations," which could be made more concrete. It would help to clarify that each Monte Carlo sample consists of a complete set of kinetic and structural parameters drawn from bootstrap-derived distributions, and that each set is used to run a full cable simulation. The Author Summary conveys this more effectively and could serve as a model for tightening the abstract. Additionally, while Table 1 lists all sampled parameters and their ranges, the Methods would benefit from a brief explicit statement of the overall workflow (sample parameters, compute resting state, simulate, classify firing behavior), and a more prominent note that each simulation uses a single parameter set applied uniformly across all compartments of the cable.

Reply: I agree that the Abstract, Author Summary, and Methods needed to describe the ensemble construction more concretely. I have revised the Abstract to state that bootstrap resampling was used to estimate uncertainty in the fitted voltage-dependent kinetic parameters, and that these uncertainty estimates were propagated through a spatially extended cable model using Monte Carlo simulations in which each sample defined a complete set of kinetic, conductance, passive, and structural parameters applied uniformly across the cable and used to run a full simulation.

I also revised the Author Summary to make the same point in less technical language. It now emphasizes that the study preserves uncertainty rather than collapsing it into a single parameter set, and that hundreds of thousands of squid axon models were simulated, each with a unique experimentally grounded parameter set.

In addition, I added a new paragraph to the Methods, immediately before the Bootstrap parameter estimation subsection, that describes the complete simulation workflow. The paragraph states that each Monte Carlo draw generated a full parameter set, including kinetic, conductance, reversal-potential, leak, and capacitance parameters from Table 1;

that the same parameter set was applied uniformly across all compartments of the cable; that the corresponding resting potential and steady-state gating variables were computed before initialization; and that each simulated voltage trace was then classified as non-firing, phasic, regular, or spontaneous according to the spike-detection criteria described in the Results. These revisions clarify both the construction of the ensemble and the distinction between ensemble simulations and a single mean-parameter simulation.

4. Scope of the sensitivity analysis outputs.
The Sobol analysis during spiking (Figure 8) quantifies sensitivity indices at two specific time points: during the action potential peak and during the sustained membrane potential. The time-varying traces in Figure 8A and 8C provide useful temporal context, but the quantitative comparison across all 22 parameters is limited to these two snapshots of membrane voltage. The authors might consider whether computing Sobol indices for additional output features, such as spike threshold, action potential width, or afterhyperpolarization, would reveal different sensitivity structures. For instance, parameters that contribute little to peak voltage variability might still strongly influence repolarization dynamics or the refractory period, which could be relevant for understanding propagation reliability. This is not essential for the current manuscript, but could provide a more complete picture of how parameter interactions shape different aspects of excitability.

Reply: I agree that feature-based Sobol analyses of spike threshold, action-potential width, afterhyperpolarization, or refractory-period-related measures would be valuable. In the present revision, I clarified the scope of Figure 8 rather than adding a new analysis. The revised Discussion now states explicitly that the t = 12 ms indices capture sensitivity during spike initiation, peak, and repolarization, whereas the t = 70 ms indices capture the sustained post-spike/depolarized state. Because the qualitative pattern of small first-order indices and large total-order indices was observed at both time points, the main conclusion, that integrated excitability is interaction-dominated, is not tied to a single scalar spike feature. I now note that feature-based Sobol decompositions would be a useful extension for future, probably extensive, work.

Reviewer #2:

I have some minor suggestions and clarification questions below, but my biggest suggestion would be to better highlight / emphasize the implication of the study, especially in the introduction and throughout the results section, because the discussion section quite clearly communicates the significance of the results contextualized against the mean model.

Reply: I agree. The Introduction and Results have been revised to bring forward the implications that were previously confined to the Discussion.
In the Introduction, the framing has been substantially restructured. A new paragraph names the specific contribution of this study in three concrete points: bootstrap-based, data-driven uncertainty estimation from the original Hodgkin–Huxley figures; the resulting population structure, including the phasic-firing dominance and the position of the canonical 1952 parameter set within the regularly firing minority; and the formal Sobol decomposition that separates first- from total-order contributions during spiking. The final

Introduction paragraph previews these three main results explicitly, so a reader reaching the Results already knows what claims the paper makes.

In the Results, several interpretive statements have been pulled forward. After the population structure and firing-rate analysis in Figures 4–6, the Results now state explicitly that the original Hodgkin–Huxley firing pattern is not the dominant behavior of the uncertainty-constrained ensemble, that the canonical parameter set lies within the regularly firing subpopulation whose mean firing rate it closely matches, and that the classical model therefore represents one structured region of parameter space rather than the full behavior of the experimentally constrained ensemble. The connection between the predominance of phasic firing and the physiological role of the squid giant axon in escape behavior is also stated in the Results rather than only in the Discussion. Together with the revised Abstract and Author Summary, which now state the ensemble reframing of the Hodgkin–Huxley model explicitly, the implications of the study should be visible at every level of the paper.

One key question I have is: would introducing variability in the kinetic parameters qualitatively change inferred conductance values or some other parameters of interesting when fitting to experimental data under the currently predominant fixed-kinetics assumption, particularly using standard optimization or inference algorithms? This can be done on simulated data, i.e., simulate with some parameter set but during optimization let the kinetic parameters be free. This could better emphasize the implications of ignoring this factor of variability for scientific inference.

Reply: I agree the question is methodologically important. To restate it in the form I take to be intended: if a ground-truth neuron is simulated with a parameter set whose kinetics differ from the canonical Hodgkin-Huxley values, and a standard fitting pipeline, genetic algorithm, multi-objective optimization, or simulation-based inference, is then used to recover the conductances under the assumption that the kinetics are fixed at the canonical values, are the recovered conductances systematically biased relative to the true conductances? And would allowing the kinetics to be free during fitting recover them correctly? The reviewer is right that this would directly demonstrate the practical consequences of ignoring kinetic variability for scientific inference, and that it can be tested cleanly on simulated data.

While I tend to agree with the reviewer's intuition that such a bias should exist, this warrants a comprehensive investigation that goes beyond adding a single simulation to the paper and may depend on the model and optimization technique selected. I have therefore added a paragraph to the Discussion (paragraph 9, immediately following the simulation-based inference paragraph) that frames this question as an explicit future direction. The paragraph names the precondition for such bias and grounds it in the present Figure 8 result, states the testable hypothesis in concrete terms, that the bias in inferred gNa and gK should scale with the deviation of true kinetics from the canonical values, and outlines the experimental design that the reviewer suggests. Recording the hypothesis here is intended to make explicit a methodological consequence of the present analysis for how detailed conductance-based models are constructed and evaluated.

high-level description of the problem / gap in the introduction is a bit unclear, e.g., in the sentence of "Highly insightful work…". Perhaps highlighting explicitly how the proposed study and approach differ from both post-hoc variability analysis and model generation would be helpful. Or is the current study considered the latter group?

Reply: I agree. The paragraph that previously opened "Highly insightful work..." has been rewritten. The Introduction now distinguishes the present study from both post-hoc variability analysis and database-style model generation: it is in the model-generation tradition in that it produces a population of behaviors, but its central addition is methodological — the population is shaped by data-driven kinetic uncertainty rather than uniform rescaling of conductances, and sensitivity analysis is built into model construction rather than applied after a model has already been selected.

The fundamental question posed in the introduction "Is neuronal excitability in conductance-based models …" has, as far as I know, been convincingly answered, in works where parameter degeneracy has been demonstrated in such HH models (the author cites many here as well).

Reply: I agree. The Introduction now poses a narrower question than the broad one of whether neuronal excitability is a population-level property, which the reviewer correctly notes has been convincingly answered by prior work. The revised text asks instead: given experimentally grounded uncertainty in the voltage-dependent kinetic parameters of the canonical Hodgkin–Huxley model, what is the structure of the resulting population of behaviors, which features of the canonical model survive that uncertainty, and to what extent is excitability governed by individual parameters rather than by their interactions?

Just to guide the reader, it would be helpful to provide a brief but explicit explanation for which values / parameters from the original equations (e.g., bottom of page 12) is being refit, ideally written with with the corresponding kinetic variable names from Table 1 instead of numerical values, before being presented with the new equations 1 & 2 (and the corresponding pairs subsequently). This would also help relating to the sensitivity curves in e.g. Figures 2 & 3.

Reply: I agree. A short paragraph has been added to the Results immediately after the original Hodgkin–Huxley rate-constant equations and before the new refit equations. The paragraph introduces the systematic naming used in Table 1 (A for amplitude prefactors, V_1/2 for half-activation voltage offsets, z for slope factors) and applies the convention to all six rate constants, identifying the fifteen fitted parameters of the n-, m-, and h-gates. The paragraph also notes that these are the parameters used as the axes of the sensitivity decompositions in Figures 2 and 3, making the link between the rate-constant section, Table 1, and the subsequent sensitivity analysis explicit.

typo in Figure 5 legend, yellow: repetitive

Reply: corrected

legend for Figure 6 A?

Reply: added

- page 19: “refitting …. revealed significant, uneven parameter uncertainty, indicating degeneracy in the voltage-dependent kinetics.” I’m not sure I understand how the propagated uncertainty indicates degeneracy? Or is it just remarking the fact that many of the randomly sampled kinetic parameter combinations result in the same qualitative behavior?

Reply: I agree the original sentence conflated two distinct senses of degeneracy. The sentence has been rewritten. The Discussion now distinguishes the structural redundancy in the rate-constant equations (different parameter combinations producing nearly identical activation and inactivation curves) from the population-level functional sense used elsewhere in the paper (different complete parameter sets producing similar firing behavior). The term "degeneracy" is now reserved for the functional sense, while the rate-equation redundancy is described directly as a property of the fitting geometry.

to clarify: is the red overlaid trace (e.g., in Fig 4) the average of all simulations, or the single simulation from the average parameter so akin to the simulation from the original HH point estimate of the parameters? The small bumps at t=5 and 20 seem to imply it’s the average of the traces, though this behavior would in general not be observed in any individual simulation? In other words, the mean of the simulations is in general not the same as simulation of the mean in such nonlinear systems. Sorry if I missed this, but it would be informative to show the mean-parameter simulation (i.e., original HH simulation) to emphasize the difference and significance, especially in light of the conclusion “This alignment suggests that the classic HH parameter set represents a special case…” (page 22).

Reply: Thank you for pointing this out. The red trace in Figure 4 is the pointwise average across all simulations, not the response of a model with average parameters and not the original Hodgkin-Huxley simulation. I have clarified this explicitly in the Results. The revised text now states that the red trace is a descriptive summary of the ensemble and should not be interpreted as a representative individual trajectory, because the system is nonlinear and the population contains multiple firing phenotypes.

I also added text to emphasize the distinction between the canonical Hodgkin-Huxley parameter set and the full uncertainty-constrained ensemble. The original Hodgkin-Huxley simulation is compared with the regularly firing subpopulation in Figure 6B, where it matches the mean firing-rate behavior of that subpopulation. The revised Results now state that the classical model remains representative of one structured region of parameter space, but does not capture the full behavior of the experimentally constrained ensemble.

- the discussion section contains many insightful interpretations and conclusions that would be helpful to already briefly state in the results section (such as the discrepancy between the original HH and population simulations here).

Reply: I agree. I have revised the Results to state the main interpretive conclusions earlier, rather than reserving them entirely for the Discussion. In particular, after presenting the population structure and firing-rate analysis in Figures 4–6, the Results now explicitly state that the original Hodgkin-Huxley firing pattern is not the dominant behavior of the uncertainty-constrained ensemble. The revised text clarifies that the canonical parameter set lies within the regularly firing subpopulation, whose mean firing-rate behavior it closely matches, and that the classical model therefore represents one structured region of parameter space rather than the full behavior of the experimentally constrained ensemble.

- line numbers and in-line figures with a caption would be very, very helpful.

Reply: Line numbers were added. I did not add inline figures because doing so would reduce their size and compromise visibility. The figures are therefore provided separately at full resolution, with all figure captions included in the manuscript.
