# Supplementary material for: Neuronal excitability and parameter variability in the Hodgkin-Huxley model
Source: PLoS Comput Biol. 2026 Jun 29;22(6):e1014458. doi: 10.1371/journal.pcbi.1014458 (PMC13336477; doi:10.1371/journal.pcbi.1014458)
Supplement: Peer review history

# Peer review: Decision Letter 0

**DOI:** 10.1371/journal.pcbi.1014458.r001

**Authors:** Hermann Cuntz, Andrea E. Martin

8 May 2026

PCOMPBIOL-D-26-00348

Neuronal excitability and parameter variability in the Hodgkin-Huxley model

PLOS Computational Biology

Dear Dr. Korngreen,

Thank you for submitting your manuscript to PLOS Computational Biology. After careful consideration, we feel that it has merit but does not fully meet PLOS Computational Biology's publication criteria as it currently stands. Therefore, we invite you to submit a revised version of the manuscript that addresses the points raised during the review process.

Please submit your revised manuscript by Jul 08 2026 11:59PM. If you will need more time than this to complete your revisions, please reply to this message or contact the journal office at ploscompbiol@plos.org. When you're ready to submit your revision, log on to https://www.editorialmanager.com/pcompbiol/ and select the 'Submissions Needing Revision' folder to locate your manuscript file.

Please include the following items when submitting your revised manuscript:

* A letter that responds to each point raised by the editor and reviewer(s). You should upload this letter as a separate file labeled 'Response to Reviewers'. This file does not need to include responses to formatting updates and technical items listed in the 'Journal Requirements' section below.

* A marked-up copy of your manuscript that highlights changes made to the original version. You should upload this as a separate file labeled 'Revised Manuscript with Track Changes'.

* An unmarked version of your revised paper without tracked changes. You should upload this as a separate file labeled 'Manuscript'.

If you would like to make changes to your financial disclosure, competing interests statement, or data availability statement, please make these updates within the submission form at the time of resubmission. Guidelines for resubmitting your figure files are available below the reviewer comments at the end of this letter.

We look forward to receiving your revised manuscript.

Kind regards,

Hermann Cuntz

Academic Editor

PLOS Computational Biology

Andrea E. Martin

Section Editor

PLOS Computational Biology

**Journal Requirements:**

1) We ask that a manuscript source file is provided at Revision. Please upload your manuscript file as a .doc, .docx, .rtf or .tex. If you are providing a .tex file, please upload it under the item type u2018LaTeX Source Fileu2019 and leave your .pdf version as the item type u2018Manuscriptu2019.

2) <carina-action-element class="ng-star-inserted">Please upload all main figures as separate Figure files in .tif or .eps format. For more information about how to convert and format your figure files please see our guidelines: </carina-action-element>

--><carina-action-element class="ng-star-inserted">https://journals.plos.org/</carina-action-element><carina-action-element class="ng-star-inserted">ploscompbiol</carina-action-element><carina-action-element class="ng-star-inserted">/s/figures</carina-action-element>

3) We have noticed that you have uploaded Supporting Information files, but you have not included a list of legends. Please add a full list of legends for your Supporting Information files after the references list.

**Reviewers' comments:**

Reviewer's Responses to Questions

**Comments to the Authors:**

**Please note here if the review is uploaded as an attachment.**

Reviewer #1: This paper makes a well-argued case for embedding parameter variability and sensitivity analysis directly into the Hodgkin-Huxley modeling pipeline, rather than treating them as post-hoc analyses. The key conceptual contribution is to shift the focus from maximal conductance variability (which has been studied extensively) to variability in the voltage-dependent kinetic parameters that define channel gating, and to ground that variability in the original experimental data via bootstrap resampling. The approach of propagating bootstrap-derived uncertainty through large-scale Monte Carlo simulations is straightforward and well-executed. The finding that phasic firing dominates the sampled population, and its connection to the squid giant axon's physiological role in escape behavior, is genuinely interesting and reframes the classic HH repetitive firing as a special case rather than the default. The Sobol decomposition showing that excitability is governed by parameter interactions rather than individual parameters is elegant and clearly presented. The paper is clearly written and the methodology is sound. I have a number of suggestions that I believe would strengthen the manuscript, outlined below roughly in order of importance.

Suggestions

1. Novelty framing and engagement with simulation-based inference literature.

The paper cites much of the relevant prior work on parameter degeneracy and robustness (Prinz, Marder, Golowasch, Goldman, etc.), but the abstract and introduction sometimes read as though the core insight, that varying HH parameters produces heterogeneous firing behaviors and that parameter interactions matter, is itself novel. Since this is well established, the framing would benefit from more clearly foregrounding what this study specifically adds: the data-driven uncertainty estimation from the original HH figures, the resulting population structure (especially phasic firing dominance), and the formal Sobol decomposition. The contributions are genuine; they just need to be distinguished more from prior work. One concrete example: the Discussion claims that kinetic parameter variability has been largely unexplored and that variability analysis is typically performed post-hoc. However, work on simulation-based inference (SBI), notably Gonçalves et al. (2020, "Training deep neural density estimators to identify mechanistic models of neural dynamics," eLife), directly investigates variability and covariance across HH parameters including kinetic parameters (see their Figure 3), and does so as part of the inference process rather than post-hoc. Acknowledging this work and related SBI literature would help position the present study's bootstrap-based approach more accurately within the field.

2. Bootstrap correlation structure.

Parameters are sampled independently from Gaussian distributions, but the authors note in the Discussion that rate-constant parameters are likely correlated. It would be valuable to report the correlation matrix from the bootstrap fits and briefly discuss which parameter pairs show strong correlations. This does not require additional simulations. The independent sampling is a reasonable choice, and the fact that structured results emerge from it is itself informative. But reporting the correlations would help readers assess the geometry of the parameter space and could motivate future work with correlated sampling.

3. Abstract and Methods clarity.

The abstract refers to "propagating uncertainty estimates through a spatially extended squid axon cable model using large-scale Monte Carlo simulations," which could be made more concrete. It would help to clarify that each Monte Carlo sample consists of a complete set of kinetic and structural parameters drawn from bootstrap-derived distributions, and that each set is used to run a full cable simulation. The Author Summary conveys this more effectively and could serve as a model for tightening the abstract. Additionally, while Table 1 lists all sampled parameters and their ranges, the Methods would benefit from a brief explicit statement of the overall workflow (sample parameters, compute resting state, simulate, classify firing behavior), and a more prominent note that each simulation uses a single parameter set applied uniformly across all compartments of the cable.

4. Scope of the sensitivity analysis outputs.

The Sobol analysis during spiking (Figure 8) quantifies sensitivity indices at two specific time points: during the action potential peak and during the sustained membrane potential. The time-varying traces in Figure 8A and 8C provide useful temporal context, but the quantitative comparison across all 22 parameters is limited to these two snapshots of membrane voltage. The authors might consider whether computing Sobol indices for additional output features, such as spike threshold, action potential width, or afterhyperpolarization, would reveal different sensitivity structures. For instance, parameters that contribute little to peak voltage variability might still strongly influence repolarization dynamics or the refractory period, which could be relevant for understanding propagation reliability. This is not essential for the current manuscript, but could provide a more complete picture of how parameter interactions shape different aspects of excitability.

Overall, this is a timely and well-executed study that advocates an important and practical shift in how biophysical models are constructed and evaluated. The suggestions above are aimed at sharpening the presentation and situating the work more precisely within the existing literature. I look forward to seeing the revised manuscript.

Reviewer #2: In this work, Korngreen reanalyze the Hodgkin-Huxley model in light of the originally ignored variability in rate constants: rate constants were visually extracted from the original figure and resampled, and the kinetic parameters are then refit to generate an empirical distribution (which was approximated by a Gaussian). Propagating this variability to conductance dynamics and firing behavior in a single and multicompartment model, the author finds a variety of behaviors, and sensitivity analysis was conducted to quantify the interacting influence of parameters on neuronal activity.

Overall, I found the paper to be very interesting. In some ways, it’s a very straightforward study, but tackles a fundamental question in a creative way. The main conclusion seems to be that kinetic parameters, which are usually treated as immutable constants in HH models, have a non-trivial influence both qualitatively (firing behavior) and quantitatively (global sensitivity analysis). Specifically, the majority of models here seem to differ from the original, mean-parameter HH behavior.

I think the work is exciting and novel, going all the way back to the channel dynamics measurements and evaluating how that variability propagate to the simulated activity. But it’s not very clearly contextualized relative to the existing literature. I have some minor suggestions and clarification questions below, but my biggest suggestion would be to better highlight / emphasize the implication of the study, especially in the introduction and throughout the results section, because the discussion section quite clearly communicates the significance of the results contextualized against the mean model.

One key question I have is: would introducing variability in the kinetic parameters qualitatively change inferred conductance values or some other parameters of interesting when fitting to experimental data under the currently predominant fixed-kinetics assumption, particularly using standard optimization or inference algorithms? This can be done on simulated data, i.e., simulate with some parameter set but during optimization let the kinetic parameters be free. This could better emphasize the implications of ignoring this factor of variability for scientific inference.

- high-level description of the problem / gap in the introduction is a bit unclear, e.g., in the sentence of “Highly insightful work…”. Perhaps highlighting explicitly how the proposed study and approach differ from both post-hoc variability analysis and model generation would be helpful. Or is the current study considered the latter group?

- The fundamental question posed in the introduction “Is neuronal excitability in conductance-based models …” has, as far as I know, been convincingly answered, in works where parameter degeneracy has been demonstrated in such HH models (the author cites many here as well).

- Just to guide the reader, it would be helpful to provide a brief but explicit explanation for which values / parameters from the original equations (e.g., bottom of page 12) is being refit, ideally written with with the corresponding kinetic variable names from Table 1 instead of numerical values, before being presented with the new equations 1 & 2 (and the corresponding pairs subsequently). This would also help relating to the sensitivity curves in e.g. Figures 2 & 3.

- typo in Figure 5 legend, yellow: repetitive

- legend for Figure 6 A?

- page 19: “refitting …. revealed significant, uneven parameter uncertainty, indicating degeneracy in the voltage-dependent kinetics.” I’m not sure I understand how the propagated uncertainty indicates degeneracy? Or is it just remarking the fact that many of the randomly sampled kinetic parameter combinations result in the same qualitative behavior?

- to clarify: is the red overlaid trace (e.g., in Fig 4) the average of all simulations, or the single simulation from the average parameter so akin to the simulation from the original HH point estimate of the parameters? The small bumps at t=5 and 20 seem to imply it’s the average of the traces, though this behavior would in general not be observed in any individual simulation? In other words, the mean of the simulations is in general not the same as simulation of the mean in such nonlinear systems. Sorry if I missed this, but it would be informative to show the mean-parameter simulation (i.e., original HH simulation) to emphasize the difference and significance, especially in light of the conclusion “This alignment suggests that the classic HH parameter set represents a special case…” (page 22).

- the discussion section contains many insightful interpretations and conclusions that would be helpful to already briefly state in the results section (such as the discrepancy between the original HH and population simulations here).

- line numbers and in-line figures with caption would be very, very helpful.

Richard Gao, PhD

Goethe University Frankfurt

**********

**Have the authors made all data and (if applicable) computational code underlying the findings in their manuscript fully available?**

The PLOS Data policy requires authors to make all data and code underlying the findings described in their manuscript fully available without restriction, with rare exception (please refer to the Data Availability Statement in the manuscript PDF file). The data and code should be provided as part of the manuscript or its supporting information, or deposited to a public repository. For example, in addition to summary statistics, the data points behind means, medians and variance measures should be available. If there are restrictions on publicly sharing data or code —e.g. participant privacy or use of data from a third party—those must be specified.

Reviewer #1: Yes

Reviewer #2: Yes

**********

PLOS authors have the option to publish the peer review history of their article (what does this mean?). If published, this will include your full peer review and any attached files.

If you choose “no”, your identity will remain anonymous but your review may still be made public.

**Do you want your identity to be public for this peer review?**  For information about this choice, including consent withdrawal, please see our Privacy Policy.

Reviewer #1: No

Reviewer #2: **Yes:**  Richard Gao

[NOTE: If reviewer comments were submitted as an attachment file, they will be attached to this email and accessible via the submission site. Please log into your account, locate the manuscript record, and check for the action link "View Attachments". If this link does not appear, there are no attachment files.]

**Figure resubmission:**

-->While revising your submission, we strongly recommend that you use PLOS’s NAAS tool (https://ngplosjournals.pagemajik.ai/artanalysis) to test your figure files. NAAS can convert your figure files to the TIFF file type and meet basic requirements (such as print size, resolution), or provide you with a report on issues that do not meet our requirements and that NAAS cannot fix.

After uploading your figures to PLOS’s NAAS tool - https://ngplosjournals.pagemajik.ai/artanalysis, NAAS will process the files provided and display the results in the "Uploaded Files" section of the page as the processing is complete. If the uploaded figures meet our requirements (or NAAS is able to fix the files to meet our requirements), the figure will be marked as "fixed" above. If NAAS is unable to fix the files, a red "failed" label will appear above. When NAAS has confirmed that the figure files meet our requirements, please download the file via the download option, and include these NAAS processed figure files when submitting your revised manuscript.

**Reproducibility:**

To enhance the reproducibility of your results, we recommend that authors of applicable studies deposit laboratory protocols in protocols.io, where a protocol can be assigned its own identifier (DOI) such that it can be cited independently in the future. Additionally, PLOS ONE offers an option to publish peer-reviewed clinical study protocols. Read more information on sharing protocols at https://plos.org/protocols?utm_medium=editorial-email&utm_source=authorletters&utm_campaign=protocols

# Author comment: Author response to Decision Letter 1

**DOI:** 10.1371/journal.pcbi.1014458.r002

19 May 2026

Attachment Submitted filename: reply5.pdf

# Peer review: Decision Letter 1

**DOI:** 10.1371/journal.pcbi.1014458.r003

**Authors:** Hermann Cuntz, Andrea E. Martin, Hermann Cuntz, Andrea E. Martin

15 Jun 2026

Dear Dr. Korngreen,

We are pleased to inform you that your manuscript 'Neuronal excitability and parameter variability in the Hodgkin-Huxley model' has been provisionally accepted for publication in PLOS Computational Biology.

Before your manuscript can be formally accepted you will need to complete some formatting changes, which you will receive in a follow up email. A member of our team will be in touch with a set of requests.

Please note that your manuscript will not be scheduled for publication until you have made the required changes, so a swift response is appreciated.

IMPORTANT: The editorial review process is now complete. PLOS will only permit corrections to spelling, formatting or significant scientific errors from this point onwards. Requests for major changes, or any which affect the scientific understanding of your work, will cause delays to the publication date of your manuscript.

Should you, your institution's press office or the journal office choose to press release your paper, you will automatically be opted out of early publication. We ask that you notify us now if you or your institution is planning to press release the article. All press must be co-ordinated with PLOS.

Thank you again for supporting Open Access publishing; we are looking forward to publishing your work in PLOS Computational Biology.

Best regards,

Hermann Cuntz

Academic Editor

PLOS Computational Biology

Andrea E. Martin

Section Editor

PLOS Computational Biology

***********************************************************

Reviewer's Responses to Questions

**Comments to the Authors:**

**Please note here if the review is uploaded as an attachment.**

Reviewer #1: The author has done a great job addressing all of my suggestions. The paper reads much more clearly now, and the specific contribution of this study is well distinguished from prior work on parameter degeneracy and simulation-based inference. I am happy to recommend acceptance.

Reviewer #2: I appreciate the author's constructive engagement with my comments. Overall, I think the substantial rewriting has better highlighted the novelty and significance of the already methodologically strong and interesting paper. I also really liked the formulation of the specific research question (L126 onwards).

I still hoped to see the results of the misspecification inference experiment (i.e., with fixed, wrong kinetic parameters). Though I agree that it deserves a more systematic treatment, and the paper can be published without it. The specific and testable hypothesis now raised in L653 is very nice though.

Richard Gao

**********

**Have the authors made all data and (if applicable) computational code underlying the findings in their manuscript fully available?**

The PLOS Data policy requires authors to make all data and code underlying the findings described in their manuscript fully available without restriction, with rare exception (please refer to the Data Availability Statement in the manuscript PDF file). The data and code should be provided as part of the manuscript or its supporting information, or deposited to a public repository. For example, in addition to summary statistics, the data points behind means, medians and variance measures should be available. If there are restrictions on publicly sharing data or code —e.g. participant privacy or use of data from a third party—those must be specified.

Reviewer #1: None

Reviewer #2: Yes

**********

PLOS authors have the option to publish the peer review history of their article (what does this mean?). If published, this will include your full peer review and any attached files.

If you choose “no”, your identity will remain anonymous but your review may still be made public.

**Do you want your identity to be public for this peer review?**  For information about this choice, including consent withdrawal, please see our Privacy Policy.

Reviewer #1: No

Reviewer #2: **Yes:**  Richard Gao

# Editor report: Acceptance letter

**DOI:** 10.1371/journal.pcbi.1014458.r004

**Authors:** Hermann Cuntz, Andrea E. Martin, Hermann Cuntz, Andrea E. Martin

PCOMPBIOL-D-26-00348R1

Neuronal excitability and parameter variability in the Hodgkin-Huxley model

Dear Dr Korngreen,

I am pleased to inform you that your manuscript has been formally accepted for publication in PLOS Computational Biology. Your manuscript is now with our production department and you will be notified of the publication date in due course.

The corresponding author will soon be receiving a typeset proof for review, to ensure errors have not been introduced during production. Please review the PDF proof of your manuscript carefully, as this is the last chance to correct any errors. Please note that major changes, or those which affect the scientific understanding of the work, will likely cause delays to the publication date of your manuscript.

Soon after your final files are uploaded, unless you have opted out, the early version of your manuscript will be published online. The date of the early version will be your article's publication date. The final article will be published to the same URL, and all versions of the paper will be accessible to readers.

For Research, Software, and Methods articles, you will receive an invoice from PLOS for your publication fee after your manuscript has reached the completed accept phase. If you receive an email requesting payment before acceptance or for any other service, this may be a phishing scheme. Learn how to identify phishing emails and protect your accounts at https://explore.plos.org/phishing.

Thank you again for supporting PLOS Computational Biology and open-access publishing. We are looking forward to publishing your work!

With kind regards,

Janani Seenivasan

PLOS Computational Biology | Carlyle House, Carlyle Road, Cambridge CB4 3DN | United Kingdom ploscompbiol@plos.org | Phone +44 (0) 1223-442824 | ploscompbiol.org | @PLOSCompBiol
